# Association of patient health education with the postoperative health related quality of life in low- intermediate recurrence risk differentiated thyroid cancer patients

**DOI:** 10.1038/s41598-025-34629-5

**Published:** 2026-01-13

**Authors:** Shuang Li, Tsz Ki Wang, Wanna Chen, Bin Li, Yuanjian Fan, Yue Chen, Daya Yang, Weiming Lv, Xiangdong Xu, Yunjian Zhang

**Affiliations:** 1 Department of Breast Surgery, the First Affiliated Hospital, Sun Yat-sen University, No.58 Zhong Shan Er Road, Guangzhou, China; 2 Department of Thyroid Surgery, the First Affiliated Hospital, Sun Yat-sen University, No.58 Zhong Shan Er Road, Guangzhou, China; 3 Clinical Trial Unit, the First Affiliated Hospital, Sun Yat-sen University, Guangzhou, China; 4 Department of Cardiology, Unit I, the First Affiliated Hospital, Sun Yat-sen University, Guangzhou, China; 5 Faculty Development Center for Health Professions Education, Sun Yat-sen University, Guangzhou, China; 6 Department of General Surgery, Unit III, the First Affiliated Hospital Nansha Division, Sun Yat-sen University, Guangzhou, China

**Keywords:** Quality of life (QoL), Health education, Thyroid neoplasms, Differentiated thyroid cancer (DTC), Postoperative recovery, Head and neck cancer, Patient education, Quality of life

## Abstract

Postoperative Differentiated Thyroid Cancer (DTC) patients with low-intermediate recurrence risk often experience anxiety and uncertainty, which adversely affect their health-related quality of life (HRQOL). Patient health education (PHE) plays a crucial role in improving HRQOL by providing information on treatment duration, management strategies and emerging therapies. This study evaluated the association between PHE and HRQOL. This cross-sectional study recruited DTC patients with low-intermediate risk of recurrence at the First Affiliated Hospital, Sun Yat-sen University, China, between October 2018 and March 2019. Eligible patients completed questionnaires assessing HRQOL and a PHE survey at 1 year postoperatively. Patients were categorized based on PHE exposure. The study cohort was derived from our previously published dataset in JAMA Surgery. Univariate and multiple regression analyses were conducted to assess the association of PHE and HRQOL. 574 eligible patients were categorized into four groups based on the type of PHE they had received: article, comic, video, and control group. Patients who received any form of PHE reported higher HRQOL scores in selected domains. Furthermore, increased frequency of PHE was associated with further improvements in HRQOL, independent of the education modality. Regression analysis identified role function, emotional function, and chilly sensation may serve as important correlates of HRQOL. Both PHE frequencies and modalities employed were positively associated with HRQOL in postoperative DTC patients under certain situations, although the observed differences were generally small in magnitude. Comprehensive and frequent PHE is associated with better postoperative recovery and HRQOL in low-intermediate recurrence risk DTC patients.

**Supplementary Information:**

The online version contains supplementary material available at 10.1038/s41598-025-34629-5.

## Introduction

The majority of differentiated thyroid cancer (DTC) patients have a favorable prognosis and long survival rate^1–4^, making their postoperative health-related quality of life (HRQOL) a critical medical concern^5,6^. However, postoperative care provided by healthcare providers is often limited, posing a significant clinical challenge in improving HRQOL for these patients.

HRQOL is a multidimensional concept encompassing physical, emotional, social, and cognitive functions^7^. Recognizing the critical role of dedicated scales in evaluating thyroid cancer survivors, the Thyroid Cancer–Specific Quality of Life Questionnaire (THYCA-QoL) has been developed as a comprehensive and reliable tool to assess HRQOL. It is often used alongside the European Organization for Research and Treatment of Cancer Quality of Life Questionnaire (EORTC QLQ-C30)^8–10^ to provide a holistic assessment.

Patient health education (PHE) is a key strategy for empowering patients to manage their health more effectively. By bridging the gap between doctors and patients, PHE fosters collaboration in addressing complex conditions. It involves disseminating scientific knowledge through various formats, such as professional materials and popular media^11^. While PHE has demonstrated effectiveness in improving public awareness and knowledge in areas like breast cancer^12^, oral health^13^, and myopia^14^, its impact on HRQOL in postoperative cancer patients remains underexplored. This study aims to address the gap by evaluating the association of PHE and the HRQOL of postoperative DTC patients.

## Materials and methods

### Setting and population

This is a cross-sectional population-based study, including DTC survivors with low-intermediate risk of recurrence from the Department of Thyroid and Breast Surgery, the First Affiliated Hospital, Sun Yat-sen University between October 2018 and March 2019. The population was derived from our previously published article “Association of Total Thyroidectomy or Thyroid Lobectomy with the Quality of Life in Patients with Differentiated Thyroid Cancer with Low to Intermediate Risk of Recurrence” in JAMA Surgery^1^. According to the 2015 American Thyroid Association Risk Stratification System for structural disease recurrence, low - intermediate risk of recurrence was defined as DTC meeting all of the following characteristics: no distant metastasis, no macroscopic invasion of the tumor into the perithyroidal soft tissues (gross extrathyroidal extension), no macroscopic residual tumor, a pathologic category of N0 or N1 without metastatic lymph nodes measuring ≥ 3 cm in greatest dimension, and follicular thyroid cancer with no more than 4 foci of vascular invasion^8^. Only patients who met the inclusion criteria of the previous study and completed a one-year postoperative HRQOL questionnaire survey were eligible for inclusion in this study. The exclusion criteria were consistent with those outlined in the previous study.

A total of 688 thyroid cancer patients were eligible to participate in the study. PHE questionnaires were distributed through email, postal mail, fax, and social media after completing postoperative HRQOL questionnaire survey in April,2020. A maximum of 3 reminders were sent via the above methods. A total of 574 questionnaires were returned, yielding a response rate of 83.4%.

This study was approved by the Clinical Research and Animal Trials Ethics Committees of the First Affiliated Hospital, Sun Yat-sen University. The research was conducted in accordance with relevant guidelines and regulations. Written informed consent was obtained from all participants and/or their legal guardians, with no financial compensation provided.

### Measures

*Socio-demographic and clinical characteristics.* Socio-demographic data were collected included age, gender, occupation, ethnicity, marital status, and education level. Clinical data included surgical methods, extent of lymph node dissection, pathological subtype of thyroid cancer, presence of microcarcinoma, and TNM stage. All participants completed the EORTC QLQ-C30, THYCA-QoL, and Hospital Anxiety and Depression Scale (HADS) questionnaires (Supplementary Method).

*Patient-health-education questionnaire.* The PHE questionnaire consisted of 3 questions: (1) “Have you read any health education materials about thyroid cancer (Yes or No)?” (2) “How many thyroid cancer-related PHE materials have you read pre- and postoperatively? (A. none; B. 0–10; C. 11–20; D. 21–30; E. >30)”. (3) “Please rank the following popular science formats (video, article, lecture, comic, venue visit, other forms) in order of your preference” (Supplementary Fig. 1–3).

### Statistical analysis

Continuous variables, including age, and domain score from EORCT QLQ-C30, THYCA-QoL and HADS were described as mean and standard deviation (SD). Independent *t*-tests or one-way ANOVA were used to compare these continuous variables between groups, including PHE parameters, sociodemographic parameters, and clinical parameters. When a significant overall effect was detected in One-way ANOVA, post-hoc multiple comparisons were conducted using the Tukey test to identify pairwise differences. Categorical variables were described as frequency and percentage. Chi-square tests or Fisher’s exact tests were used to compare categorical variables between groups, such as gender and marital status, in relation to PHE parameters. For the analysis of PHE exposure, participants were originally categorized based on predefined questionnaire options: none, 0–10, 11–20, 21–30, and > 30 articles read. Due to small sample sizes in the 21–30 and > 30 groups, these two categories were combined into a single group (> 20) to ensure statistical robustness. Multivariate linear regressions were used to estimate the relationship between the PHE parameters and domain scores from EORCT QLQ-C30, THYCA-QoL and HADS, adjusting for socio-demographic and clinical variables including gender, age, occupation, ethnicity, marital status, education level, extent of thyroidectomy and lymph node dissection, pathological diagnosis, microcarcinoma status, TNM stage and RAI treatment. All analyses were conducted using Stata/MP 14.0 software. All tests were two-sided. A *P* values less than 0.05 were considered statistically significant.

### Ethical approval

This study was approved by the Clinical Research and Animal Trials Ethics Committees of the First Affiliated Hospital, Sun Yat-sen University.

### Consent to participate

All patients provided written informed consent without financial compensation.

## Results

### Patient characteristics

Patient characteristics were summarized in Table 1. A total of 574 DTC survivors with low-intermediate recurrence risk completed the PHE questionnaires. Among them, 456 patients participated in at least on form of postoperative PHE (364 females [79.8%]; mean [SD] age, 37.65 [9.98] years), including article, comic or video, while 118 did not participated in any PHE (94 females [79.7%]; mean [SD] age, 38.25 [10.87] years). In the PHE group, 98.46% were of Han ancestry, 81.36% were married, 87.5% were employed or full-time students, and 63.16% held a bachelor’s degree or higher. A significantly higher proportion of patients in the PHE group had higher educational attainment compared to the non-PHE group (*P* <.05). Surgical methods included total thyroidectomy (45.83% in the PHE group) and lobectomy (54.17% in the PHE group). Neck lymph node dissection was not performed in 40.57% patients, while 44.08% underwent ipsilateral dissection and 15.35% underwent bilateral dissection in the PHE group. In addition, 98.68% of patients in the PHE group had a pathological diagnosis of thyroid papillary carcinoma, and 66.89% were diagnosed with microcarcinoma. The majority in the PHE group were TNM stage I (98.68%). 85.96% of patients in the PHE group did not undergo radioactive Iodine ablation (RAI). With the exception of educational level, all other baseline variables including age, sex, employment status, race, relationship status, extent of thyroidectomy, extent of neck dissection, pathological subtype, TNM stage, and RAI treatment were comparable between the PHE and non-PHE groups, with no statistically significant differences observed.

Patients who participated in PHE were more likely to have a bachelor’s degree or higher (63.16% vs. 36.84%, *P* <.001) (Table 1), and were inclined to read more PHE materials (76.3% vs. 23.7% for those who read more than 20 PHE materials, *P* <.001) (Supplementary Table 1). Patients who underwent total thyroidectomy were also more likely to have read over 20 PHE materials than those who did not (57.0% vs. 43.0%, *P* =.03) (Supplementary Table 1).

Patients who read articles were more likely to be male (21.7% vs. 6.8%, *P* =.006), to have a bachelor’s degree or higher (61.2% vs. 33.9%, *P* <.001), and to have undergone total thyroidectomy (46.8% vs. 27.1%, *P* =.005) (Supplementary Table 2).

Patients who read comics were significantly younger than those who did not (mean [SD], 36.4 [9.4] vs. 39.4 [10.8], *P* =.002). They were also more likely to be unmarried (25.2% vs. 13.4%, *P* <.001) and more likely to have a bachelor’s degree or higher (63.7% vs. 52.2%, *P* =.007) (Supplementary Table 3).

No differences in baseline characteristics were observed between patients who watched video and those who did not (all *P* >.05) (Supplementary Table 4).

### The association of socio-demographic and clinical factors, patient health education amount and modalities on EORTC QLQ-C30, THYCA-QoL, and HADS scales

**Fig. 1:**
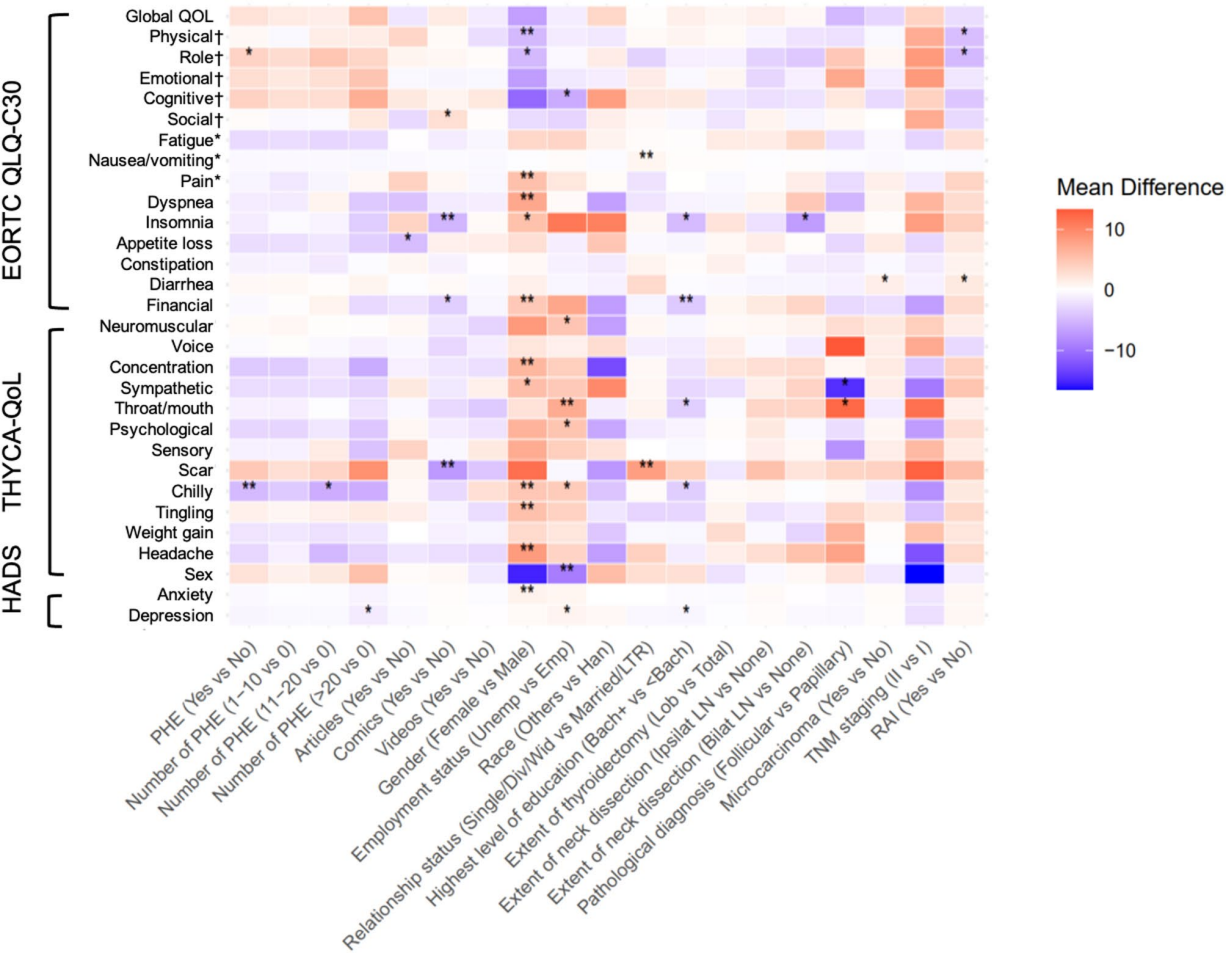
Heat map of mean differences across domains of EORTC QLQ-C30, THYCA-QoL and HADS. PHE: patient health education; RAI: radioactive Iodine ablation; Unemp: Unemployed/Retired; Emp: Employed/Full-time student; Single/Div/Wid: Single, divorced, or widowed; Married/LTR: Married or long-term relationship; Bach: Bachelor’s degree; Lob: Lobectomy; Total: Total thyroidectomy; Ipsilat LN: Ipsilateral lymph nodes dissection; Bilat LN: Bilateral lymph nodes dissection. EORTC QLQ-C30 and THYCA-QoL scores are ranged 0 to 100. HADS scores are ranged 1–21. Positive values (red) indicate higher scores in the first-listed group; negative values (blue) indicate higher scores in the reference group. †Higher scores indicate better functioning (functional domains); *Higher scores indicate more symptoms (symptom domains). Statistical significance: * *p* <.05, ** *p* <.01, *** *p* <.001.

Most socio-demographic and clinical factors were not significantly associated with the majority of EORTC QLQ-C30, THYCA-QoL and HADS domains. However, a few factors showed associations with select domains (all *P* <.05; Tables 2 and 3; Fig. 1). Specifically, being female was associated with lower scores in 9 of 15 domains of the EORTC QLQ-C30, 10 of 13 scales of the THYCA-QoL, and 1 of 2 HADS dimensions. Being unemployed was associated with lower scores in 3 of 15 EORTC QLQ-C30 domains, 5 of 13 THYCA-QoL scales, and 1 of 2 HADS dimensions. Being single was associated with lower scores in 2 of 15 EORTC QLQ-C30 domains and 1 of 13 THYCA-QoL scales, with no significant difference in HADS. Having less than a bachelor’s degree was associated with lower scores in 2 of 15 EORTC QLQ-C30 domains, 2 of 13 THYCA-QoL scales, and 1 of 2 HADS dimensions. Undergoing more extensive lymph node dissection and having a tumor size greater than 1 cm were each associated with lower scores in only 1 of 15 EORTC QLQ-C30 domains, with no significant associations observed in THYCA-QoL or HADS. Patients diagnosed with papillary cancer reported higher sympathetic syndrome score but lower throat/mouth syndrome score in THYCA-QoL (Table 3). Receiving RAI therapy was associated with lower scores in 3 of 15 EORTC QLQ-C30 domains and 1 of 13 THYCA-QoL scales, and had no significant difference in HADS. In addition, ethnicity, extent of thyroidectomy, and TNM staging were not significantly associated with any domains of the EORTC QLQ-C30, THYCA-QoL, or HADS scales.

In EORTIC QLQ-C30 scale (Table 2), patients who participated in PHE reported slightly better role function (mean [SD], 89.985 [15.847] vs. 86.299 [19.808], mean difference = 3.686, *P* =.03). Although statistically significant, the absolute difference is 3.686 points (on a 0–100 scale) may be of limited clinical significance. There was no significant difference in any HRQOL domain based on the amount of PHE materials read (0 ~ 10, 10 ~ 20, 20 ~ 30, > 30) (all *P* >.05). Patients who read articles had a lower appetite loss score (mean [SD], 2.201 [11.922] vs. 6.780 [20.323], mean difference = −4.579, *P* =.01). Patients who read comics reported slightly higher social function score (mean [SD], 94.390 [13.774] vs. 91.667 [16.415], mean difference = 2.723, *P* =.03), lower insomnia score (mean [SD], 13.290 [21.876] vs. 18.408 [22.923], mean difference = −5.118, *P* =.006) and lower financial difficulties score (mean [SD], 5.229 [13.560] vs. 8.582 [19.270], difference = −3.353, *P* =.02). Watching videos showed no significant differences in any EORTC QLQ-C30 domain (all *P* >.05).

In THYCA-QoL scale and HADS scale (Table 3), the chilly symptom score was lower (mean [SD], 6.798 [15.472] vs. 11.582 [21.954], mean difference = −4.784, *P* =.007) in patients who participated in PHE. A higher frequency of PHE participation was correlated with reduced chilly symptoms (*P* =.03) in THYCA-QoL and lower depression score (*P* =.03) in the HADS scale. Patients who read comics reported lower dissatisfaction with scars (mean [SD], 22.331 [25.583] vs. 29.478 [30.079], mean difference = −7.147, *P* =.002). For those who read articles and watch videos, there was no difference in any THYCA-QoL or HADS domains (all *P* >.05).

Multivariate linear regression analysis was performed to assess the associations of PHE on HRQOL domains, adjusting for baseline variables sociodemographic and clinical parameters (mentioned in Method-statistical analysis). PHE participation was significantly associated with improved role function (Coef [95% CI], 3.86 [0.43, 7.30], *P* =.028) and emotional function (Coef [95% CI], 3.39[0.03, 6.74], *P* =.048) in EQRCT QLQ-C30, as well as chilly symptom (Coef [95% CI], −4.16 [−7.68, −0.64], *P* =.021) in THYCA-QoL (Table 4). When considering the amount of PHE, additional associations were found with global QoL, emotional function and cognitive function in EQRCT QLQ-C30 scale; scar and chilly in THYCA-QoL scale, and depression in HADS scale (Supplementary Table 5). For article modality, appetite loss was significantly impacted in EQRCT QLQ-C30 scale (Supplementary Table 6). For comic modality, significant domains included social function, insomnia, and financial difficulties in EQRCT QLQ-C30, scar and chilly in THYCA-QoL (Supplementary Table 7). No significant associations were observed for the video modality (Supplementary Table 8).

## Discussion

With advancements in medical care, the 10-year relative survival rate of thyroid cancer has reached 98.3%^15^. Post-operative care is crucial for optimizing HRQOL. The medical paradigm has shifted from a traditional biomedical model to a biopsychosocial model, emphasizing that factors affecting postoperative HRQOL encompass not only physical health but also physical, psychological, and social factors. Consequently, HRQOL has become an important indicator for evaluating postoperative patient outcomes.

There has been a growing demand for patients to acquire medical knowledge over recent decades. PHE materials published by hospitals are typically highly professional and authoritative, making them highly appealing and influential for patients and their families. However, overly complex content will reduce engagement. We recommend implementing comprehensive and accessible PHE programs in hospitals, providing materials in various formats such as articles, comics, videos to cater to diverse learning preferences.

Previous studies on young thyroid cancer survivors have shown that, apart from impacts on major HRQOL domains, scar, headaches, anxiety, and overall psychological status significantly influenced HRQOL^16^. Meanwhile, symptoms such as fatigue, dyspnea, insomnia financial difficulties, appetite loss, constipation and diarrhea, though frequently mild in clinical presentation, remain challenging to address diseased-targeted treatment^17^. PHE serves as a valuable non-therapeutic intervention to alleviate these annoying symptoms, at least psychologically. No prior research has explored the correlation of PHE and HRQOL outcomes. Accordingly, this is the focus of our study.

Our study investigates how various PHE modalities (articles, comics, videos) and the amount of educational exposure are associated with HRQOL. We found out that highly educated individuals were more likely to participated in PHE and preferred articles over comics. Males were more likely to read articles, while younger or single individuals preferred comics. Surgical methods also influenced the choice of articles or comics. These findings suggest that tailoring PHE based on patient characteristics (age, gender, education level) might associated with higher HRQOL.

In our analysis, different PHE modalities showed distinct associations with HRQOL outcomes. Overall, participation in PHE, as well as the amount and modality of engagement, was associated with improvements in certain domains and reductions in symptom domains, suggesting a potential positive impact on patients’ overall QoL. However, given the small magnitude of difference, the clinical relevance of these associations should be interpreted with caution.

Multi-regression analysis revealed significant improvements in role function (Coef [95% CI], 3.86 [0.43, 7.30], *P* =.03) and emotional function (Coef [95% CI], 3.39[0.03, 6.74], *P* =.049) in EORTC QLQ-C30 scale, and chilly symptom (Coef [95% CI], −4.16 [−7.68, −0.64], *P* =.02) in THYCA-QoL scale, indicating that PHE may help patients better manage daily activities and emotional challenges. Participation in PHE may reduce stress, annoyance, anxiety or other negative emotions, enabling patients to focus more on treatment and rehabilitation. Improved chilly symptoms may result in better compliance on medication adherence and regular thyroid function monitoring, reducing hypothyroidism-related issues^18^. However, one point we should not overlook is that the coefficients are all modest on a 0–100 scale, which suggests that the clinical significance may be limited.

Increased PHE participation was associated with significant improvement in global QoL (Coef [95% CI], 1.52 [0.03, 3.01], *P* =.046), emotional function (Coef [95% CI], 1.72 [0.39, 3.05], *P* =.011) and cognitive functions (Coef [95% CI], 1.76 [0.02, 3.50], *P* =.048) on the EORTC QLQ-C30 scale, scar satisfaction (Coef [95% CI], 2.43 [0.19, 4.67], *P* =.033) and chilly symptoms (Coef [95% CI], −1.53 [−2.93, −0.13], *P* =.032) of THYCA-QoL scale, and depression on the HADS scale (Coef [95% CI], −0.34, [−0.62, −0.06], *P* =.017). As patients become more engaged in PHE, their mood, memory, and attention improve, likely due to increased awareness of emotional management and proactive problem solving. Similarly, the clinical relevance of these findings should be interpreted with caution, given that the absolute values of the regression coefficients are relatively small.

Analysis of PHE modalities revealed that articles was positively associated with appetite scores on the EORTC QLQ-C30 (Coef [95% CI], −4.11 [−7.75, −0.46], *P* =.027); while comics were associated with better outcome in social function (Coef [95% CI], 2.96 [0.44, 5.48], *P* =.021), insomnia (Coef [95% CI], −4.98 [−8.66, −1.31], *P* =.008), and financial difficulties (Coef [95% CI], −3.18 [−5.87, −0.49], *P* =.02) on the EORTC QLQ-C30 scale, as well as scar satisfaction (Coef [95% CI], −10.08 [−14.58, −5.58], *P* <.001) and chilly symptoms (Coef [95% CI], −2.91 [−5.77, −0.06], *P* =.045) on the THYCA-QoL. A study suggested that guiding readers to relevant passages and providing plain language summaries make reading medical papers easier and give readers more confidence to approach these papers^19^. Similarly, our research suggests that comics, with their accessible format, may be more strongly associated with improved HRQOL. It is also important to interpret these findings with caution, as some of the absolute values of the regression coefficients are relatively small and the clinical significance of these associations may be limited. We recommend transforming traditional medical knowledge into innovative, easy-to-understand formats like graphics, comics, and videos to enhance accessibility and engagement.

The developers of the THYCA-QoL scale suggested that higher educational level enable patients to choose better healthcare providers, ask more informed questions, and better understand their conditions, potentially reducing anxiety and depression^17,20^. In our regression models, after adjusting for baseline characteristics, including educational level, positive associations of PHE and HRQOL remain significant. This indicates that the relationship between PHE and HRQOL persists even after adjusting for patient background characteristics and healthcare access, suggesting an independent association. Also, major complaints, such as scar, anxiety and depression in younger age patients, or insomnia in Asian population are alleviated through PHE^16,20^.

Our study is the first large-scale investigation to examine the association between PHE and postoperative HRQOL in thyroid cancer patients. It offers a comprehensive multi-faceted analysis of PHE, considering both its quantity and modalities. We established a WeChat official account to regularly disseminate diverse PHE materials and created patient groups for online communication with medical teams, enhancing doctor-patient trust and addressing postoperative concerns^21^. Using both global HRQOL (EORTC QLQ-C30) and the disease-specific scale (THYCA-QoL), supplemented by emotional scale HADS, we provided a comprehensive HRQOL assessment of patient outcomes. Adjusting for baseline characteristics further strengthened the credibility of our findings. Since each form of PHE is associated with the HRQOL in different dimensions, we believe that each form of PHE holds value, and it will be more efficient to conduct integrated forms of PHE.

This study has several limitations that should be acknowledged. First, it was single-center designed and conducted at a leading Chinese hospital with experienced surgeons, which may introduce population bias. Second, the short follow-up period of 1-year post-surgery is another limitation, as HRQOL improvements in role and emotional functioning, fatigue, pain, and dyspnea may evolve over longer periods^22^. Future studies should evaluate the long-term impacts of regular, tailored, and systematic PHE through prospective, multicenter clinical trials. Third, PHE exposure was self-reported, which may be subject to recall bias. Participants may have inaccurately reported their engagement with educational materials, potentially affecting the reliability of the observed associations. Fourth, PHE exposure was categorized based on predefined questionnaire options rather than treated as a continuous variable. While this approach was intended to facilitate participant recall and encourage questionnaire completion, it may have limited the level of detail in the analysis. Future studies may consider using continuous measures or data-driven cut points (e.g., quartiles) to enhance analytical precision and interpretability. Fifth, selection bias may have occurred, as only patients who completed the follow-up survey were included in the analysis. These individuals may differ systematically from non-respondents in ways that influence both PHE engagement and HRQOL outcomes. Sixth, the cross-sectional design of the study limits the ability to infer causality. And we didn’t have pre-treatment QoL data for comparison. Without longitudinal data, the temporal relationship between PHE exposure and HRQOL outcomes cannot be established. This also restricts our capacity to attribute differences directly to PHE interventions. It is possible that patients with better HRQOL were more likely to engage in PHE, rather than PHE leading to improved HRQOL. Therefore, reverse causality and residual confounding cannot be ruled out, and the observed associations should be interpreted with caution. Seventh, although we adjusted for a wide range of socio-demographic and clinical variables in our multivariate regression analyses, the possibility of residual confounding cannot be fully excluded. Unmeasured factors such as baseline HRQOL, patient motivation, health-seeking behavior, and access to healthcare services may have influenced both the likelihood of engaging in PHE and the reported HRQOL outcomes. These variables were not captured in our dataset and may partially account for the observed associations. Future studies should consider incorporating these factors to better isolate the independent effects of PHE on postoperative recovery and quality of life. Finally, although the results revealed statistical differences, the effect sizes were modest across some domains. Given the small magnitude of these differences, the findings may not be clinically significant. For example, receiving PHE was statistically associated with a 3.77-point increase in Role function domain, the magnitude of this effect was modest relative to the 100-point scale. Therefore, the clinical relevance of this finding remains uncertain. Further investigation should be carried out to determine the clinical implications of these results.

## Conclusions

Increased participation in PHE, through both greater engagement and diverse modalities, is associated the HRQOL of thyroid patients after surgery. Notable improvements were observed in role function, emotional function, social function, appetite loss, insomnia, financial difficulties, chilly sensations, scar satisfaction, and depression. Therefore, comprehensive and tailored PHE programs are highly recommended as they are associated with better postoperative recovery and HRQOL in thyroid cancer patients.

**Table 1:** Demographics of questionnaires respondents (no PHE & PHE).

	Total (*n* = 574)	No PHE (*n* = 118)	PHE (*n* = 456)	*P* values
Age	37.77 (10.16)	38.25(10.87)	37.65 (9.98)	0.74*
Gender (n, %)				> 0.99
Male	116 (20.21)	24 (20.34)	92 (20.18)	
Female	458 (79.79)	94 (79.66)	364 (79.82)	
Employment status (n, %)				0.14
Employed/Full-time student	496 (86.41)	97 (82.20)	399 (87.50)	
Unemployed/Retired	78 (13.59)	21 (17.80)	57 (12.50)	
Race (n, %)				> 0.99
Han	565 (98.43)	116 (98.31)	449 (98.46)	
Others	9 (1.57)	2 (1.69)	7 (1.54)	
Relationship status (n, %)				0.24
Married or long-term relationship	461 (80.31)	90 (76.27)	371 (81.36)	
Single, divorced, or widowed	113 (19.69)	28 (23.73)	85 (18.64)	
Highest level of education (n, %)				< 0.001
Below bachelor’s degree	239 (41.64)	71 (60.17)	168 (36.84)	
Bachelor’s degree or above	335 (58.36)	47 (39.83)	288 (63.16)	
Extent of thyroidectomy (n, %)				0.35
Total thyroidectomy	257 (44.77)	48 (40.68)	209 (45.83)	
Lobectomy	317 (55.23)	70 (59.32)	247 (54.17)	
Extent of neck dissection (n, %)				0.90
No neck dissection	232 (40.42)	47 (39.83)	185 (40.57)	
Ipsilateral lymph nodes dissection	252 (43.90)	51 (43.22)	201 (44.08)	
Bilateral lymph nodes dissection	90 (15.68)	20 (16.95)	70 (15.35)	
Pathological diagnosis (n, %)				0.67
Papillary	566 (98.61)	116 (98.31)	450 (98.68)	
Follicular	8 (1.39)	2 (1.69)	6 (1.32)	
Microcarcinoma (n, %)				0.51
No	380 (66.20)	75 (63.56)	305 (66.89)	
Yes	194 (33.80)	43 (36.44)	151 (33.11)	
TNM staging (n, %)				> 0.99
I	567 (98.78)	117 (99.15)	450 (98.68)	
II	7 (1.22)	1 (0.85)	6 (1.32)	
RAI (n, %)				0.651
No	496 (86.41)	104 (88.14)	392 (85.96)	
Yes	78 (13.59)	14 (11.86)	64 (14.04)	

PHE: patient health education; RAI: radioactive Iodine ablation.

*Not conform to bivariate normal distribution. Non-parametric test is performed and data were uniformly described by mean (SD).

**Table 2:** EQRCT QLQ-C30 scores in different groups.

		Global QOL		Physical†		Role†		Emotional†	Cognitive†	Social†		Fatigue*		Nausea/vomiting*
PHE (*P* value, [Mean difference])		0.19 [2.426]		0.70 [0.61]		0.03 [3.686]		0.10 [2.848]	0.09 [3.734]	0.88 [0.227]		0.22 [−2.56]		0.14 [−0.419]
No		70.692(17.287)		84.068 (16.073)		86.299 (19.808)		73.870 (17.120)	74.153 (23.930)	92.938 (16.653)		20.810 (22.764)		0.565 (4.321)
Yes		73.118(18.431)		84.678 (15.360)		89.985 (15.847)		76.718 (16.327)	77.887 (20.941)	93.165 (14.707)		18.250 (19.467)		0.146 (2.205)
Number of PHE		0.19		0.61		0.14		0.16	0.13	0.58		0.67		0.46
0		70.692 (17.287)		84.068 (16.073)		86.299 (19.808)		73.870 (17.120)	74.153 (23.930)	92.938 (16.653)		20.810 (22.764)		0.565 (4.321)
1–10		72.305 (20.000)		83.615 (15.719)		89.303 (16.592)		75.746 (17.143)	76.866 (21.850)	92.537 (14.986)		18.463 (19.516)		0.166 (2.351)
11–20		72.163 (17.390)		85.485 (15.498)		91.135 (14.578)		76.478 (17.374)	76.950 (21.516)	92.671 (16.106)		18.046 (20.033)		0.236 (2.807)
> 20		75.731 (16.614)		85.556 (14.544)		89.766 (16.067)		78.728 (13.169)	80.848 (18.316)	94.883 (12.170)		18.129 (18.830)		0.000 (0.000)
Articles (*P* value, [Mean difference])		0.50[−1.71]		0.11[3.437]		0.70[0.902]		0.84[−0.47]	0.53[1.89]	0.21[−2.632]		0.98[−0.062]		0.33[−0.371]
No		74.153 (17.280)		81.469 (16.144)		88.418 (16.157)		76.554 (16.338)	75.424 (21.962)	95.480 (12.314)		18.832 (20.452)		0.565 (4.340)
Yes		72.443 (18.325)		84.906 (15.398)		89.320 (16.869)		76.084 (16.554)	77.314 (21.594)	92.848 (15.388)		18.770 (20.185)		0.194 (2.539)
Comics (*P* value, [Mean difference])		0.26[1.716]		0.77[0.375]		0.63[0.674]		0.63[−0.676]	0.60[0.943]	0.03[2.723]		0.46[−1.252]		0.26[−0.264]
No		71.704 (19.164)		84.353 (15.226)		88.868 (17.293)		76.493 (15.464)	76.617 (21.697)	91.667 (16.415)		19.444 (20.289)		0.373 (3.513)
Yes		73.420 (17.330)		84.728 (15.754)		89.542 (16.350)		75.817 (17.409)	77.560 (21.579)	94.390 (13.774)		18.192 (20.126)		0.109 (1.906)
Videos (*P* value, [Mean difference])		0.62[−1.33]		0.31[−2.316]		0.92[0.249]		0.81[−0.586]	0.54[1.957]	0.95[0.13]		0.84[−0.61]		0.25[−0.476]
No		73.833 (15.431)		86.667 (13.669)		89.000 (16.361)		76.667 (14.773)	75.333 (23.143)	93.000 (17.846)		19.333 (21.626)		0.667 (4.714)
Yes		72.503 (18.466)		84.351 (15.657)		89.249 (16.841)		76.081 (16.687)	77.290 (21.485)	93.130 (14.846)		18.723 (20.074)		0.191 (2.517)
Gender (*P* value, [Mean difference])		< 0.001[−6.675]		0.003[−4.81]		0.01[−4.857]		< 0.001[−6.954]	< 0.001[−10.668]	0.11[−2.502]		0.11[3.365]		0.81[−0.069]
Male		77.945 (16.676)		88.391 (14.015)		93.103 (13.382)		81.681 (14.956)	85.632 (16.433)	95.115 (11.634)		16.092 (18.086)		0.287 (3.095)
Female		71.270 (18.356)		83.581 (15.717)		88.246 (17.419)		74.727 (16.614)	74.964 (22.250)	92.613 (15.844)		19.457 (20.657)		0.218 (2.692)
Employment status (*P* value, [Mean difference])		0.59[−1.201]		0.49[−1.312]		0.85[−0.392]		0.44[−1.558]	0.03[−5.914]	0.11[−2.917]		0.16[3.493]		0.51[0.225]
Employed/Full-time student		72.782 (17.998)		84.731 (15.449)		89.281 (16.714)		76.344 (16.115)	77.923(21.171)	93.515 (14.172)		18.302 (20.133)		0.202 (2.587)
Unemployed/Retired		71.581 (19.621)		83.419 (15.854)		88.889 (17.339)		74.786 (18.945)	72.009 (23.798)	90.598 (20.027)		21.795 (20.452)		0.427 (3.774)
Race (*P* value, [Mean difference])		0.58[3.36]		0.76[−1.615]		0.79[1.537]		0.84[−1.15]	0.26[8.194]	0.79[1.347]		0.88[0.992]		0.80[−0.236]
Han		72.566 (18.236)		84.578 (15.461)		89.204 (16.798)		76.150 (16.523)	76.991 (21.667)	93.097 (15.102)		18.761 (20.075)		0.236 (2.797)
Others		75.926 (17.403)		82.963 (18.592)		90.741 (16.897)		75.000 (17.180)	85.185 (17.568)	94.444 (16.667)		19.753 (28.207)		0.000 (0.000)
Relationship status (*P* value, [Mean difference])		0.95[0.118]		0.83[0.355]		0.08[−3.115]		0.38[1.528]	0.37[2.044]	0.76[0.488]		0.91[0.25]		0.005[0.813]
Married or long-term relationship		72.596 (18.612)		84.483 (15.415)		89.841 (16.295)		75.832 (16.582)	76.717 (21.568)	93.022 (15.072)		18.727 (19.777)		0.072 (1.552)
Single, divorced, or widowed		72.714 (16.564)		84.838 (15.893)		86.726 (18.522)		77.360 (16.270)	78.761 (21.850)	93.510 (15.339)		18.977 (21.907)		0.885 (5.383)
Highest level of education (*P* value, [Mean difference])		0.40[1.297]		0.53[0.823]		0.48[−1.013]		0.75[−0.45]	0.30[1.899]	0.66[−0.559]		0.97[0.071]		0.50[0.16]
Below bachelor’s degree		71.862 (18.572)		84.073 (15.812)		89.819 (16.342)		76.395 (17.597)	76.011 (21.630)	93.445 (14.536)		18.735 (19.664)		0.139 (2.156)
Bachelor’s degree or above		73.159 (17.962)		84.896 (15.283)		88.806 (17.107)		75.945 (15.728)	77.910 (21.610)	92.886 (15.529)		18.806 (20.593)		0.299 (3.145)
Extent of thyroidectomy (*P* value, [Mean difference])		0.63[0.739]		0.79[0.353]		0.50[−0.953]		0.59[0.759]	0.34[−1.739]	0.16[−1.775]		0.33[1.667]		0.43[0.185]
Total thyroidectomy		72.211 (18.201)		84.358 (15.613)		89.754 (16.824)		75.713 (16.322)	78.080 (20.504)	94.099 (13.692)		17.856 (18.677)		0.130 (2.079)
Lobectomy		72.950 (18.245)		84.711 (15.425)		88.801 (16.769)		76.472 (16.694)	76.341 (22.486)	92.324 (16.151)		19.523 (21.346)		0.315 (3.232)
Extent of neck dissection		0.75		0.61		0.07		0.18	0.52	0.60		0.40		0.73
No neck dissection		71.983 (19.993)		85.201 (14.912)		91.164 (15.186)		77.514 (15.824)	78.376 (21.107)	92.816 (16.663)		17.577 (20.000)		0.287 (3.088)
Ipsilateral lymph nodes dissection		73.247 (16.732)		84.392 (15.827)		88.095 (17.203)		74.735 (17.491)	76.190 (21.959)	93.783 (13.673)		19.136 (20.163)		0.132 (2.100)
Bilateral lymph nodes dissection		72.500 (17.509)		83.333 (16.122)		87.407 (19.079)		76.481 (15.278)	76.481 (22.051)	92.037 (14.829)		20.864 (20.792)		0.370 (3.514)
Pathological diagnosis (*P* value, [Mean difference])		0.44[−4.98]		0.71[−2.082]		0.44[4.586]		0.22[7.302]	0.79[2.076]	0.91[0.64]		0.77[−2.139]		0.81[−0.236]
Papillary		72.688 (18.276)		84.582 (15.549)		89.164 (16.868)		76.031 (16.561)	77.091 (21.732)	93.110 (15.157)		18.806 (20.292)		0.236 (2.795)
Follicular		67.708 (12.939)		82.500 (11.785)		93.750 (8.626)		83.333 (11.785)	79.167 (11.785)	93.750 (12.400)		16.667 (11.878)		0.000 (0.000)
Microcarcinoma (*P* value, [Mean difference])		0.07[−2.892]		0.75[−0.441]		0.64[0.7]		0.39[−1.256]	0.16[−2.682]	0.99[0.013]		0.81[−0.419]		0.15[−0.351]
No		73.596 (18.247)		84.702 (15.793)		88.991 (17.528)		76.557 (16.444)	78.026 (21.902)	93.114 (15.461)		18.918 (20.526)		0.351 (3.406)
Yes		70.704 (18.040)		84.261 (14.936)		89.691 (15.259)		75.301 (16.675)	75.344 (21.000)	93.127 (14.447)		18.499 (19.578)		0.000 (0.000)
TNM staging (*P* value, [Mean difference])		0.60[3.615]		0.24[6.961]		0.18[8.495]		0.18[8.495]	0.64[3.88]	0.23[6.966]		0.70[−2.939]		0.82[−0.235]
I		72.575 (18.245)		84.468 (15.550)		89.124 (16.850)		76.029 (16.546)	77.072 (21.717)	93.034 (15.187)		18.812 (20.286)		0.235 (2.792)
II		76.190 (16.265)		91.429 (8.357)		97.619 (6.299)		84.524 (12.199)	80.952 (11.501)	0.000 (0.000)		15.873 (10.843)		0.000 (0.000)
RAI (*P* value, [Mean difference])		. 30[−2.313]		0.02[−4.477]		0.02[−4.843]		0.40[−1.682]	0.14[−3.937]	0.15[−2.669]		0.28[2.669]		0.43[−0.269]
No		72.933 (17.824)		85.161 (14.852)		89.886 (15.897)		76.361 (16.179)	77.655 (21.021)	93.481 (15.059)		18.414 (20.074)		0.269 (2.984)
Yes		70.620 (20.532)		80.684 (18.751)		85.043 (21.251)		74.679 (18.583)	73.718 (24.985)	90.812 (15.348)		21.083 (20.928)		0.000 (0.000)

	Pain*		Dyspnea		Insomnia		Appetite loss		Constipation		Diarrhea		Financial	
PHE (*P* value, [mean difference])	0.68 [−0.768]		0.58 [−1.246]		0.59[−1.243]		0.09[−2.327]		0.32[−0.881]		0.47[0.531]		0.84[−0.337]	
No	11.441(17.528)		18.644(24.085)		16.667(24.941)		4.520(16.831)		1.977(10.026)		0.565(4.321)		7.062(15.624)	
Yes	10.673(18.039)		17.398(21.300)		15.424(21.842)		2.193(11.904)		1.096 (8.045)		1.096(7.736)		6.725(16.790)	
Number of PHE	0.76		0.36		0.60		0.26		0.48		0.74		0.35	
0	11.441(17.528)		18.644(24.085)		16.667(24.941)		4.520(16.831)		1.977(10.026)		0.565(4.321)		7.062(15.624)	
1–10	9.867 (17.019)		17.247(21.368)		16.418(22.881)		2.322(12.253)		1.161 (7.730)		1.161(7.730)		7.131(16.633)	
11–20	10.757(19.327)		19.622(22.562)		15.839(20.924)		2.837(13.504)		0.473 (5.614)		0.709(6.259)		8.038(19.478)	
> 20	11.988(18.218)		14.912(19.377)		13.158(21.090)		1.170 (8.791)		1.754(10.719)		1.462(9.292)		4.386(12.939)	
Articles (*P* value, [Mean difference])	0.12[3.885]		0.16[−4.252]		0.24[3.623]		0.01[−4.579]		0.50[0.794]		0.87[−0.159]		0.41[−1.873]	
No	7.345 (13.229)		21.469(22.963)		12.429(17.396)		6.780(20.323)		0.565 (4.340)		1.130(6.084)		8.475(17.057)	
Yes	11.230(18.352)		17.217(21.739)		16.052(22.993)		2.201(11.922)		1.359 (8.839)		0.971(7.287)		6.602(16.491)	
Comics (*P* value, [Mean difference])	0.63[0.718]		0.44[−1.415]		0.006[−5.118]		0.24[1.278]		0.22[−0.87]		0.45[0.452]		0.02[−0.353]	
No	10.448(18.322)		18.408(22.741)		18.408(22.923)		1.990(11.366)		1.741 (9.840)		0.746(6.407)		8.582(19.270)	
Yes	11.166(17.588)		16.993(21.122)		13.290(21.876)		3.268(14.418)		0.871 (7.088)		1.198(7.778)		5.229(13.560)	
Videos (*P* value, [Mean difference])	0.84[−0.551]		0.73[−1.11]		0.91[0.379]		0.45[1.466]		0.96[−0.061]		0.72[−0.379]		0.81[−0.59]	
No	11.333(19.178)		18.667(19.237)		15.333(25.386)		1.333 (9.428)		1.333 (9.428)		1.333(9.428)		7.333(19.390)	
Yes	10.782(17.817)		17.557(22.135)		15.712(22.228)		2.799(13.383)		1.272 (8.403)		0.954(6.925)		6.743(16.267)	
Gender (*P* value, [Mean difference])	0.004[5.291]		0.001[7.359]		0.03[5.245]		0.05[2.627]		0.56[0.521]		0.10[1.237]		0.008[4.554]	
Male	6.609 (12.014)		11.782(18.260)		11.494(20.194)		0.575 (6.190)		0.862 (6.896)		0.000(0.000)		3.161(10.748)	
Female	11.900(18.994)		19.141(22.487)		16.739(22.943)		3.202(14.272)		1.383 (8.850)		1.237(8.007)		7.715(17.604)	
Employment status (*P* value, [Mean difference])	0.35[2.056]		0.90[0.341]		< 0.001[11.034]		0.49[−1.114]		0.34[−0.984]		0.46[−0.648]		< 0.001[7.468]	
Employed/Full-time student	10.551(17.331)		17.608(21.587)		14.180(21.359)		2.823(13.438)		1.411 (9.003)		1.075(7.563)		5.780(14.610)	
Unemployed/Retired	12.607(21.352)		17.949(23.839)		25.214(26.960)		1.709(10.606)		0.427 (3.774)		0.427(3.774)		13.248(24.817)	
Race (*P* value, [Mean difference])	0.96[0.285]		0.37[−6.647]		0.17[10.41]		0.27[4.811]		0.65[−1.298]		0.68[−1.003]		0.22[−6.903]	
Han	10.826(17.928)		17.758(21.954)		15.516(22.483)		2.596(12.908)		1.298 (8.554)		1.003(7.224)		6.903 (16.653)	
Others	11.111(18.634)		11.111(16.667)		25.926 (22.222)		7.407 (22.222)		0.000 (0.000)		0.000(0.000)		0.000 (0.000)	
Relationship status (*P* value, [Mean difference])	0.31[−1.916]		0.44[−1.78]		0.77[0.678]		0.78[−0.388]		0.27[0.98]		< 0.001[3.178]		0.67[−0.746]	
Married or long-term relationship	11.208(18.250)		18.004(22.117)		15.546(22.129)		2.748(13.267)		1.085 (8.002)		0.362(4.648)		6.941 (16.448)	
Single, divorced, or widowed	9.292 (16.508)		16.224(20.947)		16.224(24.035)		2.360(12.374)		2.065(10.238)		3.540(12.881)		6.195 (16.990)	
Highest level of education (*P* value, [Mean difference])	0.98[0.037]		0.66[−0.818]		0.01[−4.679]		0.54[−0.68]		0.70[0.277]		0.72[−0.22]		0.009[−3.652]	
Below bachelor’s degree	10.809 (17.898)		18.131 (22.588)		18.410 (23.972)		3.068 (13.999)		1.116 (7.401)		1.116 (7.401)		8.926 (18.443)	
Bachelor’s degree or above	10.846 (17.967)		17.313 (21.397)		13.731 (21.204)		2.388 (12.408)		1.393 (9.195)		0.896 (7.007)		5.274 (14.886)	
Extent of thyroidectomy (*P* value, [Mean difference])	0.76[−0.469]		0.71[−0.679]		0.22[2.322]		0.58[0.699]		0.11[1.139]		0.59[−0.326]		0.57[0.795]	
Total thyroidectomy	11.089 (19.015)		18.029 (22.994)		14.397 (22.732)		2.335 (12.279)		0.649 (5.474)		1.167 (7.983)		6.355 (16.359)	
Lobectomy	10.620 (17.013)		17.350 (20.975)		16.719 (22.286)		2.944 (13.719)		1.788 (10.286)		0.841 (6.441)		7.150 (16.710)	
Extent of neck dissection	0.79		0.24		0.046		0.46		0.52		0.46		0.21	
No neck dissection	10.560 (18.925)		16.523 (20.338)		17.672 (23.600)		2.011 (11.429)		1.580 (9.428)		1.437 (9.193)		5.460 (14.839)	
Ipsilateral lymph nodes dissection	10.648 (16.771)		17.460 (21.952)		15.608 (21.327)		3.439 (14.776)		1.323 (8.827)		0.661 (5.527)		7.275 (17.476)	
Bilateral lymph nodes dissection	12.037 (18.534)		21.111 (25.208)		10.741 (22.250)		2.222 (12.034)		0.370 (3.514)		0.741 (4.941)		8.889 (17.877)	
Pathological diagnosis (*P* value, [Mean difference])	0.69[−2.533]		0.50[−5.227]		0.90[1.002]		0.56[−2.709]		0.67[−1.296]		0.70[−1.001]		0.65[−2.665]	
Papillary	10.866 (17.992)		17.727 (21.947)		15.665 (22.481)		2.709 (13.175)		1.296 (8.547)		1.001 (7.218)		6.832 (16.606)	
Follicular	8.333 (12.599)		12.500 (17.252)		16.667 (25.198)		0.000 (0.000)		0.000 (0.000)		0.000 (0.000)		4.167 (11.785)	
Microcarcinoma (*P* value, [Mean difference])	0.42[1.289]		0.66[0.845]		0.92[0.194]		0.15[1.675]		0.62[−0.373]		0.03[1.364]		0.13[−2.218]	
No	10.395 (18.063)		17.368 (21.579)		15.614 (22.755)		2.105 (11.674)		1.404 (8.951)		0.526 (5.389)		7.544 (17.673)	
Yes	11.684 (17.659)		18.213 (22.519)		15.808 (22.039)		3.780 (15.458)		1.031 (7.517)		1.890 (9.710)		5.326 (14.000)	
TNM staging (*P* value, [Mean difference])	0.85[−1.323]		0.45[6.232]		0.34[8.231]		0.59[−2.704]		0.69[−1.293]		0.71[−0.999]		0.28[−6.878]	
I	10.847 (17.955)		17.578 (21.675)		15.579 (22.380)		2.704 (13.164)		1.293 (8.539)		0.999 (7.211)		6.878 (16.629)	
II	9.524 (16.265)		23.810 (37.090)		23.810 (31.706)		0.000 (0.000)		0.000 (0.000)		0.000 (0.000)		0.000 (0.000)	
RAI (*P* value, [Mean difference])	. 11[3.54]		0.21[2.971]		0.13[4.11]		0.25[1.855]		0.34[0.995]		0.04[1.825]		0.13[3.018]	
No	10.349 (17.516)		17.204 (21.181)		15.121 (21.343)		2.419 (12.480)		1.142 (8.263)		0.739 (6.133)		6.384 (16.33)	
Yes	13.889 (20.175)		20.175 (25.893)		19.231 (28.689)		4.274 (16.435)		2.137 (9.818)		2.564 (11.732)		9.402 (17.734)	

PHE: patient health education; RAI: radioactive Iodine ablation.

EQRCT QLQ-C30 scores are ranged 0 to 100.

Data are expressed as mean (SD) unless otherwise specified. Mean differences of parameters with more than 2 groups were compared pairwise. Such comparisons are not presented in this table. See statistics significance for those with P value < 0.05 in Fig. 1.

†Higher scores indicate better functioning (functional domains); *Higher scores indicate more symptoms (symptom domains).

**Table 3:** THYCA-QoL and HADS scores in different groups.

	Neuromuscular		Voice		Concentration		Sympathetic		Throat/mouth		Psychological		Sensory		Scar
PHE (*P* value, [mean difference])	0.83[0.352]		0.89[−0.268]		0.08[−3.616]		0.25[−2.463]		0.59[−0.956]		0.13[−2.767]		0.62[−0.986]		0.11[4.574]
No	16.290(16.351)		12.147(20.976)		20.904(23.183)		18.362(22.264)		17.891(18.549)		22.175(20.156)		18.785(19.798)		22.034(28.318)
Yes	16.642(15.540)		11.879(18.936)		17.288(19.304)		15.899(20.255)		16.935(16.601)		19.408(17.032)		17.800(19.563)		26.608(27.842)
Number of PHE	0.98		0.97		0.14		0.70		0.80		0.35		0.11		0.08
0	16.290(16.351)		12.147(20.976)		20.904(23.183)		18.362(22.264)		17.891(18.549)		22.175(20.156)		18.785(19.798)		22.034(28.318)
1–10	16.971(16.142)		12.272(18.523)		17.330(20.470)		16.003(18.622)		16.860(15.796)		19.279(18.364)		17.828(20.583)		24.710(27.943)
11–20	16.391(15.802)		11.820(20.073)		19.031(19.475)		16.194(23.226)		17.809(19.194)		20.508(17.006)		20.449(19.555)		25.532(28.070)
> 20	16.374(14.195)		11.257(18.351)		15.058(16.736)		15.351(19.221)		15.984(14.486)		18.275(14.492)		14.474(17.248)		31.287(27.078)
Articles (*P* value, [Mean difference])	0.77[0.626]		0.84[−0.552]		0.72[−0.998]		0.50[1.913]		0.86[−0.427]		0.78[0.699]		0.17[3.693]		0.82[0.902]
No	16.008(13.572)		12.429(20.898)		18.927(19.193)		14.689(15.501)		17.514(16.914)		19.350(16.770)		14.689(14.870)		24.859(30.066)
Yes	16.634(15.932)		11.877(19.191)		17.929(20.320)		16.602(21.204)		17.087(17.034)		20.049(17.857)		18.382(20.046)		25.761(27.757)
Comics (*P* value, [Mean difference])	0.19[−1.737]		0.10[−2.696]		0.30[−1.757]		0.37[−1.54]		0.07[−2.628]		0.40[−1.257]		0.75[−0.528]		0.002[−7.147]
No	17.496(16.227)		13.371(20.156)		18.968(20.295)		17.226(21.702)		18.532(18.214)		20.647(18.238)		18.284(19.922)		29.478(30.079)
Yes	15.759(15.196)		10.675(18.566)		17.211(20.102)		15.686(19.765)		15.904(15.804)		19.390(17.292)		17.756(19.340)		22.331(25.583)
Videos (*P* value, [Mean difference])	0.19[−3.027]		0.68[−1.168]		0.47[−2.156]		0.70[1.175]		0.15[−3.629]		0.48[−1.851]		0.53[1.829]		0.33[−4.015]
No	19.333(19.545)		13.000(21.373)		20.000(21.296)		15.333(20.437)		20.444(17.149)		21.667(20.960)		16.333(20.617)		29.333(29.845)
Yes	16.306(15.275)		11.832(19.170)		17.844(20.096)		16.508(20.728)		16.815(16.976)		19.816(17.412)		18.162(19.512)		25.318(27.797)
Gender (*P* value, [Mean difference])	< 0.001[8.522]		0.28[2.172]		0.004[6.032]		0.01[5.435]		0.18[2.383]		< 0.001[6.489]		< 0.001[7.076]		< 0.001[11.64]
Male	9.770 (10.273)		10.201(15.350)		13.218(17.379)		12.069(18.203)		15.230(16.677)		14.799(14.500)		12.356(16.019)		16.379(23.050)
Female	18.292(16.360)		12.373(20.234)		19.250(20.686)		17.504(21.149)		17.613(17.074)		21.288(18.247)		19.432(20.171)		28.020(28.640)
Employment status (*P* value, [Mean difference])	0.01[4.893]		0.59[1.273]		0.10[4.108]		0.07[4.506]		0.001[7.045]		0.02[4.948]		0.08[4.141]		0.88[−0.525]
Employed/Full-time student	15.905(15.112)		11.761(19.172)		17.473(19.632)		15.793(20.110)		16.174(15.578)		19.304(16.723)		17.440(18.861)		25.739(27.514)
Unemployed/Retired	20.798(18.554)		13.034(20.570)		21.581(23.279)		20.299(23.821)		23.219(23.429)		24.252(22.839)		21.581(23.587)		25.214(30.947)
Race (*P* value, [Mean difference])	0.20[−6.799]		0.65[2.927]		0.06[−12.674]		0.16[9.672]		0.85[−1.099]		0.30[−6.185]		0.72[2.405]		0.44[−7.263]
Han	16.676(15.722)		11.888(19.085)		18.230(20.243)		16.254(20.456)		17.148(16.961)		20.074(17.742)		17.965(19.582)		25.782(28.037)
Others	9.877 (12.963)		14.815(33.793)		5.556 (11.785)		25.926(32.394)		16.049(20.869)		13.889(17.180)		20.370(21.695)		18.519(24.216)
Relationship status (*P* value, [Mean difference])	0.68[0.671]		0.59[−1.086]		0.81[0.505]		0.75[0.693]		0.59[0.952]		0.46[−1.367]		> 0.99[−0.01]		0.006[8.075]
Married or long-term relationship	16.438(15.219)		12.148(18.925)		17.932(19.999)		16.269(20.600)		16.944(16.439)		20.246(17.630)		18.004(19.319)		24.078(26.579)
Single, divorced, or widowed	17.109(17.570)		11.062(21.083)		18.437(21.054)		16.962(21.127)		17.896(19.213)		18.879(18.197)		17.994(20.791)		32.153(32.407)
Highest level of education (*P* value, [Mean difference])	0.65[−0.604]		0.39[−1.419]		0.25[−1.963]		0.12[−2.718]		0.02[−3.386]		0.58[−0.84]		0.78[−0.46]		0.09[4.072]
Below bachelor’s degree	16.922(15.904)		12.762(19.412)		19.177(20.112)		17.992(22.545)		19.107(17.557)		20.467(17.713)		18.271(19.885)		23.291(28.197)
Bachelor’s degree or above	16.318(15.565)		11.343(19.320)		17.214(20.241)		15.274(19.209)		15.721(16.486)		19.627(17.770)		17.811(19.419)		27.363(27.736)
Extent of thyroidectomy (*P* value, [Mean difference])	0.76[0.412]		0.39[1.412]		0.21[2.119]		0.20[−2.234]		0.88[−0.215]		0.87[0.24]		0.98[−0.048]		0.48[−1.668]
Total thyroidectomy	16.342(15.215)		11.154(17.081)		16.861(19.626)		17.639(21.095)		17.250(16.919)		19.844(16.574)		18.029(19.745)		26.589(26.637)
Lobectomy	16.754(16.097)		12.566(21.024)		18.980(20.624)		15.405(20.330)		17.035(17.105)		20.084(18.650)		17.981(19.510)		24.921(29.038)
Extent of neck dissection	0.85		0.85		0.27		0.36		0.06		0.40		0.76		0.11
No neck dissection	16.140(15.739)		12.284(22.176)		16.379(19.553)		15.230(20.406)		15.086(15.972)		19.217(17.522)		17.313(19.452)		22.989(25.732)
Ipsilateral lymph nodes dissection	16.755(15.188)		11.971(18.007)		19.048(20.957)		16.601(20.588)		18.519(17.265)		21.098(18.120)		18.651(20.138)		28.307(30.364)
Bilateral lymph nodes dissection	17.160(17.081)		10.926(14.815)		19.444(19.553)		18.889(21.666)		18.519(18.473)		18.796(17.199)		17.963(18.567)		25.185(26.114)
Pathological diagnosis (*P* value, [Mean difference])	0.60[2.915]		0.05[13.25]		0.92[0.729]		0.049[−14.525]		0.04[12.206]		0.72[−2.301]		0.27[−7.693]		0.72[3.549]
Papillary	16.529(15.737)		11.749(19.250)		18.021(20.277)		16.608(20.753)		16.961(16.796)		20.009(17.807)		18.110(19.692)		25.618(28.071)
Follicular	19.444(12.944)		25.000(23.570)		18.750(13.909)		2.083 (5.893)		29.167(27.176)		17.708(12.148)		10.417 (8.626)		29.167(21.362)
Microcarcinoma (*P* value, [Mean difference])	0.18[1.876]		0.40[1.44]		0.34[1.702]		0.43[1.433]		0.38[−1.307]		0.62[0.775]		0.43[1.356]		0.13[3.793]
No	15.936(15.644)		11.447(18.790)		17.456(20.850)		15.921(20.479)		17.573(17.762)		19.715 (18.111)		17.544(19.238)		24.386(27.535)
Yes	17.812(15.763)		12.887(20.431)		19.158(18.842)		17.354(21.112)		16.266(15.431)		20.490(17.010)		18.900(20.307)		28.179(28.729)
TNM staging (*P* value, [Mean difference])	0.49[4.115]		0.33[7.202]		0.62[−3.792]		0.23[−9.377]		0.07[11.581]		0.30[−6.967]		0.43[5.879]		0.24[12.58]
I	16.520(15.669)		11.846(19.244)		18.078(20.255)		16.520(20.769)		16.990(16.796)		20.062(17.753)		17.931(19.487)		25.514(27.945)
II	20.635(18.624)		19.048(27.936)		14.286(14.996)		7.143 (8.909)		28.571(29.297)		13.095(15.853)		23.810(28.637)		38.095(29.991)
RAI (*P* value, [Mean difference])	0.46[1.431]		0.26[−2.683]		0.12[3.861]		0.05[5]		0.54[1.276]		0.19[2.846]		0.49[1.668]		0.11[5.409]
No	16.375 (15.647)		12.298 (19.960)		17.507 (19.957)		15.726 (20.162)		16.958 (17.065)		19.590 (17.476)		17.776 (19.228)		24.933 (27.768)
Yes	17.806 (16.051)		9.615 (14.839)		21.368 (21.471)		20.726 (23.445)		18.234 (16.700)		22.436 (19.244)		19.444 (21.891)		30.342 (29.023)

		Chilly		Tingling		Weight gain		Headache		Sex		Anxiety		Depression	
PHE (*P* value, [mean difference])		0.007 [−4.784]		0.55[1.165]		0.49[−1.781]		0.27[−2.703]		0.33[2.413]		0.35[−0.327]		0.06[−0.676]	
No		11.582(21.954)		8.192(17.935)		20.056(24.707)		23.729(25.450)		33.333(23.061)		3.932(3.510)		3.678(3.748)	
Yes		6.798 (15.472)		9.357(19.015)		18.275(24.656)		21.026(23.202)		35.746(24.038)		3.605(3.387)		3.002(3.320)	
Number of PHE (P value)		0.03		0.91		0.92		0.32		0.36		0.27		0.03	
0		11.582(21.954)		8.192(17.935)		20.056(24.707)		23.729(25.450)		33.333(23.061)		3.932(3.510)		3.678(3.748)	
1–10		7.960 (16.421)		8.955(19.649)		18.408(25.571)		22.720(23.761)		34.494(24.581)		3.801(3.575)		3.204(3.469)	
11–20		5.910 (14.521)		9.456(19.650)		17.967(23.744)		18.810(23.387)		35.225(21.739)		3.716(3.510)		3.227(3.502)	
> 20		5.848 (14.871)		9.942(17.135)		18.421(24.327)		20.760(21.901)		38.596(25.697)		3.123(2.829)		2.368(2.711)	
Articles (*P* value, [Mean difference])		0.84[0.487]		0.60[1.346]		> 0.99[−0.003]		0.59[−1.763]		0.94[0.247]		0.71[−0.177]		0.50[0.315]	
No		7.345 (15.247)		7.910(18.916)		18.644(23.384)		23.164(22.535)		35.028(22.679)		3.831(3.445)		3.424(3.405)	
Yes		7.832 (17.307)		9.256(18.788)		18.641(24.819)		21.401(23.828)		35.275(23.991)		3.654(3.412)		3.109(3.423)	
Comics (*P* value, [Mean difference])		0.06[−2.668]		0.58[−0.867]		0.64[−0.962]		0.28[−2.134]		0.78[0.562]		0.49[0.198]		0.72[0.104]	
No		9.204 (18.648)		9.577(19.232)		19.154(25.602)		22.722(23.476)		34.950(23.647)		3.567(3.395)		3.086(3.368)	
Yes		6.536 (15.534)		8.71(18.415)		18.192(23.828)		20.588(23.859)		35.512(24.044)		3.765(3.430)		3.190(3.469)	
Videos (*P* value, [Mean difference])		0.29[2.682]		0.38[−2.427]		0.84[−0.758]		0.45[−2.649]		0.66[−1.552]		0.65[−0.227]		0.93[−0.043]	
No		5.333(14.062)		11.333(21.936)		19.333(27.013)		24.000(23.367)		36.667(21.560)		3.880(3.438)		3.180(3.415)	
Yes		8.015(17.351)		8.906(18.471)		18.575(24.446)		21.351(23.724)		35.115(24.061)		3.653(3.413)		3.137(3.424)	
Gender (*P* value, [Mean difference])		0.008[4.711]		0.007[5.304]		0.21[3.195]		0.001[8.325]		< 0.001[−15.245]		0.003[1.037]		0.22[0.436]	
Male		4.023 (11.759)		4.885(11.840)		16.092(24.665)		14.943(21.689)		47.414(24.138)		2.845(3.136)		2.793(3.256)	
Female		8.734 (18.087)		10.189(20.042)		19.287(24.638)		23.268(23.895)		32.169(22.780)		3.882(3.451)		3.229(3.458)	
Employment status (*P* value, [Mean difference])		0.04[4.347]		0.10[3.791]		0.47[2.166]		0.22[3.573]		0.002[−9.142]		0.05[0.809]		0.03[0.905]	
Employed/Full-time student		7.191(16.543)		8.602(17.788)		18.347(24.469)		21.102(23.461)		36.492(23.620)		3.563(3.214)		3.018(3.291)	
Unemployed/Retired		11.538(19.976)		12.393(24.090)		20.513(25.893)		24.675(25.016)		27.350(23.870)		4.372(4.437)		3.923(4.086)	
Race (*P* value, [Mean difference])		0.47[−4.143]		0.783[−1.738]		0.64[−3.887]		0.39[−6.875]		0.49[5.579]		> 0.99[−0.006]		0.64[0.534]	
Han		7.847 (17.173)		9.145(18.856)		18.702(24.679)		21.690(23.766)		35.162(23.708)		3.673(3.414)		3.133(3.407)	
Others		3.704 (11.111)		7.407(14.699)		14.815(24.216)		14.815(17.568)		40.741(32.394)		3.667(3.536)		3.667(4.387)	
Relationship status (*P* value, [Mean difference])		0.90[0.228]		0.14[−2.094]		0.87[−0.438]		0.11[3.982]		0.27[2.756]		0.93[−0.033]		0.14[−0.528]	
Married or long-term relationship		7.737 (16.894)		9.689(18.974)		18.727(24.548)		20.797(22.757)		34.707(22.641)		3.679(3.443)		3.245(3.459)	
Single, divorced, or widowed		7.965 (17.970)		6.785(17.906)		18.289(25.194)		24.779(27.014)		37.463(28.219)		3.646(3.298)		2.717(3.233)	
Highest level of education (*P* value, [Mean difference])		0.02[−3.395]		0.08[−2.779]		0.79[−0.56]		0.56[−1.174]		0.20[2.566]		0.37[−0.26]		0.03[−0.647]	
Below bachelor’s degree		9.763 (18.774)		10.739(20.060)		18.968(23.131)		22.269(23.598)		33.752(25.287)		3.824(3.669)		3.519(3.581)	
Bachelor’s degree or above		6.368 (15.666)		7.960 (17.768)		18.408(25.719)		21.095(23.770)		36.318(22.731)		3.564(3.218)		2.872(3.279)	
Extent of thyroidectomy (*P* value, [Mean difference])		0.74[0.469]		0.52[1.009]		0.15[2.988]		0.50[1.354]		0.34[−1.931]		0.37[−0.255]		0.79[−0.076]	
Total thyroidectomy		7.523 (18.031)		8.560(18.523)		16.991 (23.220)		20.833 (23.616)		36.316 (25.425)		3.813 (3.545)		3.183 (3.352)	
Lobectomy		7.992 (16.323)		9.569(19.019)		19.979 (25.719)		22.187 (23.760)		34.385 (22.477)		3.558 (3.302)		3.107 (3.479)	
Extent of neck dissection		0.87		0.50		0.61		0.17		0.94		0.28		0.33	
No neck dissection		7.471 (15.868)		10.201(20.488)		19.253 (26.006)		19.540 (22.396)		35.345 (24.140)		3.487 (3.343)		3.103 (3.424)	
Ipsilateral lymph nodes dissection		8.201 (18.432)		8.201(16.930)		18.915 (23.603)		22.354 (24.142)		34.921 (23.375)		3.929 (3.506)		3.329 (3.525)	
Bilateral lymph nodes dissection		7.407 (16.405)		8.889(19.223)		16.296 (24.091)		24.719 (25.390)		35.926 (24.595)		3.433 (3.312)		2.711 (3.088)	
Pathological diagnosis (*P* value, [Mean difference])		0.93[0.559]		0.61[3.431]		0.46[6.449]		0.36[7.692]		0.79[2.282]		0.73[−0.428]		0.67[−0.523]	
Papillary		7.774 (17.130)		9.069(18.820)		18.551 (24.758)		21.475 (23.799)		35.218 (23.889)		3.678 (3.416)		3.148 (3.435)	
Follicular		8.333 (15.430)		12.500(17.252)		25.000 (15.430)		29.167 (11.785)		37.500 (21.362)		3.250 (3.370)		2.625 (2.200)	
Microcarcinoma (*P* value, [Mean difference])		0.46[−1.113]		0.28[1.8]		0.95[0.133]		0.94[−0.158]		0.45[−1.597]		0.35[0.285]		0.60[0.16]	
No		8.158 (17.814)		8.509(18.301)		18.596 (24.914)		21.636 (24.746)		35.789 (24.571)		3.576 (3.359)		3.087 (3.428)	
Yes		7.045 (15.612)		10.309(19.705)		18.729 (24.204)		21.478 (21.522)		34.192 (22.363)		3.861 (3.516)		3.247 (3.410)	
TNM staging (*P* value, [Mean difference])		0.23[−7.878]		0.54[−4.409]		0.58[5.233]		0.18[−12.207]		0.07[−16.402]		0.16[−1.838]		0.10[−2.168]	
I		7.878 (17.179)		9.171(18.854)		18.577 (24.664)		21.731 (23.734)		35.450 (23.848)		3.695 (3.423)		3.168 (3.432)	
II		0.000 (0.000)		4.762(12.599)		23.810 (25.198)		9.524 (16.265)		19.048 (17.817)		1.857 (1.773)		1.000 (0.816)	
RAI (*P* value, [Mean difference])		. 37[1.875]		0.15[3.297]		0.47[2.166]		0.27[3.214]		0.67[−1.229]		0.10[0.69]		0.10[0.683]	
No		7.527 (16.865)		8.669 (18.316)		18.347 (24.652)		21.145 (22.883)		35.417 (23.834)		3.579 (3.311)		3.048 (3.336)	
Yes		9.402 (18.530)		11.966 (21.471)		20.513 (24.753)		24.359 (28.260)		34.188 (24.010)		4.269 (3.973)		3.731 (3.884)	

PHE: patient health education; RAI: radioactive Iodine ablation.

THYCA-QoL scores are ranged 0 to 100. HADS scores are ranged 1–21.

Data are expressed as mean (SD) unless otherwise specified. Mean differences of parameters with more than 2 groups were compared pairwise. Such comparisons are not presented in this table. See statistics significance for those with P value < 0.05 in Fig. 1.

**Table 4:** Multifactor linear regression analysis of PHE and EQRCT QLQ-C30, THYCA-QoL, HADS.

Variables	Coef.	95% CI	*P* values
Global QOL	2.47	(−1.28, 6.23)	0.20
Physical†	0.55	(−2.65, 3.75)	0.74
Role†	3.86	(0.43, 7.30)	0.03
Emotional†	3.39	(0.03, 6.74)	0.049
Cognitive†	3.66	(−0.73, 8.04)	0.10
Social†	0.26	(−2.87, 3.38)	0.87
Fatigue*	−2.51	(−6.71, 1.68)	0.24
Nausea/vomiting*	−0.39	(−0.96, 0.19)	0.18
Pain*	−0.92	(−4.61, 2.76)	0.62
Dyspnea	−1.46	(−5.99, 3.07)	0.53
Insomnia	−0.14	(−4.71, 4.43)	0.95
Appetite loss	−2.31	(−5.02, 0.40)	0.10
Constipation	−0.96	(−2.73, 0.80)	0.29
Diarrhea	0.73	(−0.73, 2.19)	0.32
Financial	0.74	(−2.59, 4.08)	0.66
Neuromuscular	0.56	(−2.61, 3.73)	0.73
Voice	0.18	(−3.84, 4.20)	0.93
Concentration	−3.26	(−7.39, 0.87)	0.12
Sympathetic	−1.77	(−6.02, 2.47)	0.41
Throat/mouth	−0.04	(−3.54, 3.46)	0.98
Psychological	−2.93	(−6.57, 0.71)	0.11
Sensory	−1.07	(−5.13, 2.98)	0.60
Scar	3.69	(−1.95, 9.33)	0.20
Chilly	−4.16	(−7.68, −0.64)	0.02
Tingling	1.82	(−2.02, 5.66)	0.35
Weight gain	−1.75	(−6.90, 3.39)	0.50
Headache	−2.05	(−6.91, 2.81)	0.41
Sex	2.09	(−2.60, 6.78)	0.28
Anxiety	−0.32	(−1.02, 0.38)	0.37
Depression	−0.59	(−1.30, 0.12)	0.10

The baseline factors were adjusted including sex, age, occupation, ethnicity, marital status, educational level, extent of thyroidectomy and lymph node dissection, paraffin pathological diagnosis, paraffin diagnosed as microcarcinoma, TNM staging.

†Higher scores indicate better functioning (functional domains); *Higher scores indicate more symptoms (symptom domains).

## Supplementary Information

Below is the link to the electronic supplementary material.

Supplementary Material 1

## Data Availability

The data that support the findings of this study are available from Dr. Yunjian Zhang, Dr. Shuang Li and Dr. Wanna Chen, but restrictions apply to the availability of these data, therefore, they are not publicly available. Data are however available from the authors upon reasonable request and with permission of Dr. Yunjian Zhang, Dr. Shuang Li, Dr. Wanna Chen and author(s) from the article “Association of Total Thyroidectomy or Thyroid Lobectomy With the Quality of Life in Patients With Differentiated Thyroid Cancer With Low to Intermediate Risk of Recurrence” (doi:10.1001/jamasurg.2021.6442).

## Abbreviations

DTC: Differentiated thyroid cancer
HRQOL: Health-related quality of life
PHE: Patient health education
QoL: Quality of Life
THYCA-QoL: Thyroid Cancer–Specific Quality of Life Questionnaire
EORTC QLQ-C30: European Organization for Research and Treatment of Cancer Quality of Life Questionnaire
HADS: Hospital Anxiety and Depression Scale
RAI: Radioactive Iodine ablation
