# Supplementary material for: Association of patient health education with the postoperative health related quality of life in low- intermediate recurrence risk differentiated thyroid cancer patients
Source: Sci Rep. 2026 Jan 13;16:4565. doi: 10.1038/s41598-025-34629-5 (PMC12868796; doi:10.1038/s41598-025-34629-5)
Supplement: Supplementary file 1 — Supplementary Material 1 [file 41598_2025_34629_MOESM1_ESM.docx]

**Supplementary Method.** Introduction of HRQOL questionnaires

*Tumor core HRQOL (EORTC QLQ-C30).* EORTC QLQ-C30 is a core scale that can be administered to patients with any type of cancer. It consists of 1 global QoL domain, 5 functional domains (physical function, role function, emotional function, cognitive function, social function), 3 symptom domains (fatigue, nausea and vomiting, pain), and 6 single items (dyspnea, sleep disturbance, appetite loss, constipation, diarrhea, and financial difficulties) [^1^](#_ENREF_1)^,^[^2^](#_ENREF_2). EORTC QLQ-C30 has a total of 30 items, and items 29 and 30 are divided into 7 levels, which are counted as 1 to 7 points according to answer options. Other items are divided into 4 levels: “not at all”, “a little”, “quite a bit” and “very much”, and scored 1 to 4 points respectively. For functional domains and global QoL, the higher the score, the better the HRQOL. For the symptom domains, the higher the score, the worse the HRQOL. The score of the domains can be obtained by summing the scores of the items included in each domain and then dividing it by the number of items included. To make the scores in various domains comparable with each other, the linear transformation method is further used to convert the rough scores into standardized scores (0-100 points).

*Thyroid cancer-specific HRQOL (THYCA-QoL)*. THYCA-QoL is a method-developed questionnaire that can be used in conjunction with EORTC QLQ-C30 instrument to assess major aspects of the HRQOL of thyroid cancer survivors [^3^](#_ENREF_3). The questionnaire includes 24 items, assessing 7 scales (neuromuscular, voice, concentration, sympathetic, throat/mouth problems, psychological and sensory problems) and six individual items (scar, chilly, tingling hands/feet, gained weight, headaches, interested in sex). Each item is scored on a 4-point scale, from 1 as "nothing at all" to 4 as "very", and scores are linearly converted to 0-100 with higher score means more uncomfortable and a worse QoL. The combination of THYCA-QoL and the EORTC QLQ-C30 has been successfully applied to evaluate the HRQOL of patients with thyroid cancer after surgery [^4-8^](#_ENREF_4).

*Psychological distress (HADS).* HADS is used to assess psychological distress [^9^](#_ENREF_9). The scale has two dimensions (anxiety and depression), and each dimension has 7 questions. The options for each question are divided into 4 levels (0-3 points). The scores of each question are added together to obtain the total score. When diagnosing anxiety, all odd-numbered item scores must be added to the total score, and when diagnosing depression, all even-numbered item scores must be added to the total score. The total of 0-7 indicates no anxiety or depression, a score of 8-10 indicates possible or "critical" anxiety or depression, and a score of 11-20 indicates there may be significant anxiety or depression.

**References**

**1.** Sprangers M, Cull A, Groenvold M, et al. The European Organization for Research and Treatment of Cancer approach to developing questionnaire modules: an update and overview. EORTC Quality of Life Study Group. 1998;7(4):291-300.

**2.** Aaronson NK, Ahmedzai S, Bergman B, et al. The European Organization for Research and Treatment of Cancer QLQ-C30: a quality-of-life instrument for use in international clinical trials in oncology. *Journal of the National Cancer Institute.* Mar 3 1993;85(5):365-376.

**3.** Husson O, Haak H, Mols F, et al. Development of a disease-specific health-related quality of life questionnaire (THYCA-QoL) for thyroid cancer survivors. 2013;52(2):447-454.

**4.** Lan Y, Luo Y, Zhang M, et al. Quality of Life in Papillary Thyroid Microcarcinoma Patients Undergoing Radiofrequency Ablation or Surgery: A Comparative Study. *Frontiers in endocrinology.* 2020;11:249.

**5.** Lan Y, Jin Z, Zhang Y, et al. Factors associated with health-related quality of life in papillary thyroid microcarcinoma patients undergoing radiofrequency ablation: a cross-sectional prevalence study. 2020;37(1):1174-1181.

**6.** Goldfarb M, Casillas J. Thyroid Cancer-Specific Quality of Life and Health-Related Quality of Life in Young Adult Thyroid Cancer Survivors. *Thyroid : official journal of the American Thyroid Association.* Jul 2016;26(7):923-932.

**7.** Husson O, Nieuwlaat WA, Oranje WA, Haak HR, van de Poll-Franse LV, Mols F. Fatigue among short- and long-term thyroid cancer survivors: results from the population-based PROFILES registry. *Thyroid : official journal of the American Thyroid Association.* Oct 2013;23(10):1247-1255.

**8.** Husson O, Haak HR, Buffart LM, et al. Health-related quality of life and disease specific symptoms in long-term thyroid cancer survivors: a study from the population-based PROFILES registry. *Acta oncologica.* Feb 2013;52(2):249-258.

**9.** Zigmond AS, Snaith RP. The hospital anxiety and depression scale. *Acta psychiatrica Scandinavica.* Jun 1983;67(6):361-370.

**Supplementary Index**

**Supplementary Figure 1-3** Original patient health education article, comic and video created by our center (with English translation)

**Supplementary Table 1-4** Demographics of questionnaire respondents in different amount of patient health education, and in different modalities

**Supplementary Table 5-8** Multifactor linear regression analysis of the amount of patient health education, and in different modalities in EQRCT QLQ-C30, THYCA-QoL and HADS scales

**Supplementary Figure 1.** **Example of patient health education: an article**

(authored by Dr. Weiming Lv and Dr. Jie Li from our Department of Thyroid and Breast Surgery)


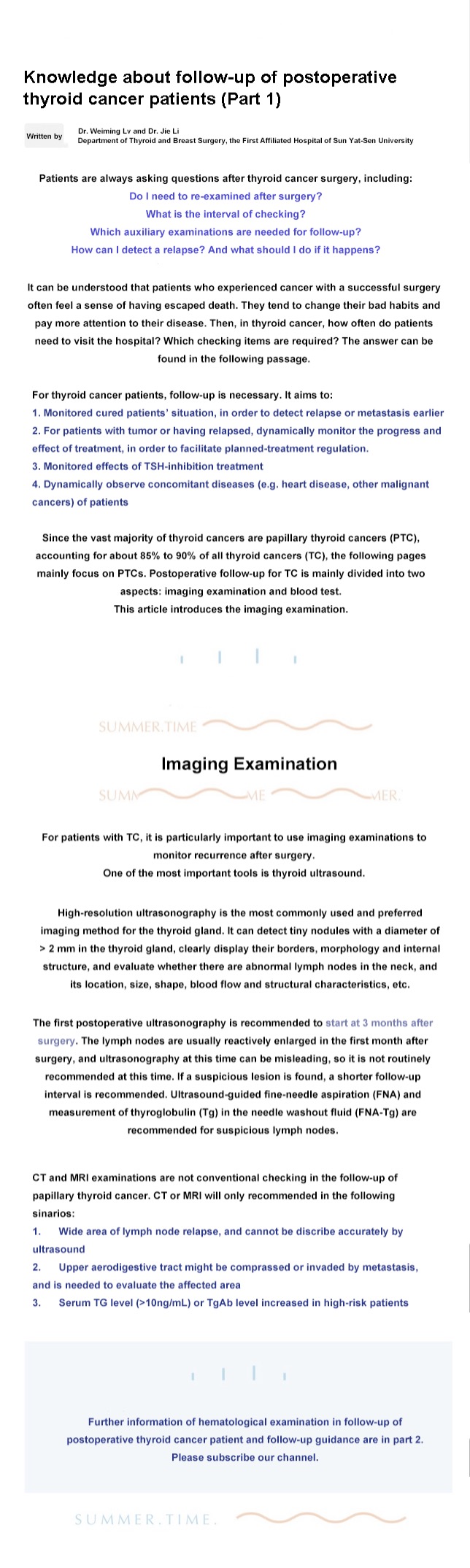


Full article link (Chinese version): https://mp.weixin.qq.com/s/0TXei5caiskVWXYtp8f8CA

English translation of the sample article:

Knowledge about follow-up of postoperative thyroid cancer patients (Part 1)

Written by Dr. Weiming Lv and Dr. Jie Li

Department of Thyroid and Breast Surgery, the First Affiliated Hospital of Sun Yat-Sen University

Patients are always asking questions after thyroid cancer surgery, including:

Do I need to re-examined after surgery?

What is the interval of checking?

Which auxiliary examinations are needed for follow-up?

How can I detect a relapse? And what should I do if it happens?

It can be understood that patients who experienced cancer with a successful surgery often feel a sense of having escaped death. They tend to change their bad habits and pay more attention to their disease. Then, in thyroid cancer, how often do patients need to visit the hospital? Which checking items are required? The answer can be found in the following passage.

For thyroid cancer patients, follow-up is necessary. It aims to:

1. Monitored cured patients’ situation, in order to detect relapse or metastasis earlier

2. For patients with tumor or having relapsed, dynamically monitor the progress and effect of treatment, in order to facilitate planned-treatment regulation.

3. Monitored effects of TSH-inhibition treatment

4. Dynamically observe concomitant diseases (e.g., heart disease, other malignant cancers) of patients

Since the vast majority of thyroid cancers are papillary thyroid cancers (PTC), accounting for about 85% to 90% of all thyroid cancers (TC), the following pages mainly focus on PTCs. Postoperative follow-up for TC is mainly divided into two aspects: imaging examination and blood test. This article introduces the imaging examination.

**Imaging Examination**

For patients with TC, it is particularly important to use imaging examinations to monitor recurrence after surgery. One of the most important tools is thyroid ultrasound.

High-resolution ultrasonography is the most commonly used and preferred imaging method for the thyroid gland. It can detect tiny nodules with a diameter of > 2 mm in the thyroid gland, clearly display their borders, morphology and internal structure, and evaluate whether there are abnormal lymph nodes in the neck, and its location, size, shape, blood flow and structural characteristics, etc.

The first postoperative ultrasonography is recommended to start at 3 months after surgery. The lymph nodes are usually reactively enlarged in the first month after surgery, and ultrasonography at this time can be misleading, so it is not routinely recommended at this time. If a suspicious lesion is found, a shorter follow-up interval is recommended. Ultrasound-guided fine-needle aspiration (FNA) and measurement of thyroglobulin (Tg) in the needle washout fluid (FNA-Tg) are recommended for suspicious lymph nodes.

CT and MRI examinations are not conventional checking in the follow-up of papillary thyroid cancer. CT or MRI will only recommended in the following sinarios:

1. Wide area of lymph node relapse, and cannot be discribe accurately by ultrasound
2. Upper aerodigestive tract might be comprassed or invaded by metastasis, and is needed to evaluate the affected area
3. Serum TG level (>10ng/mL) or TgAb level increased in high-risk patients

Further information of hematological examination in follow-up of postoperative thyroid cancer patient and follow-up guidance are in part 2. Please follow us.

**Supplementary Figure 2.** **Example of patient health education: a comic** (original work by Dr. YunJian Zhang, listed corresponding author, from our Department of Thyroid and Breast Surgery, created in collaboration with a comic studio), used with permission under a CC BY open access license.


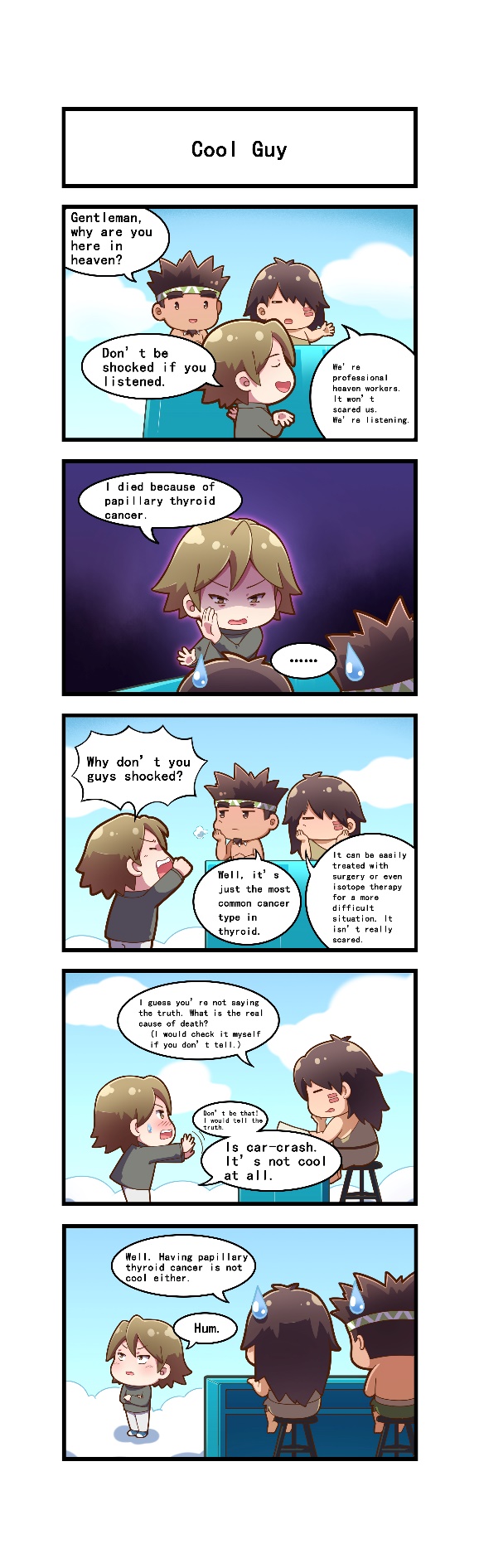

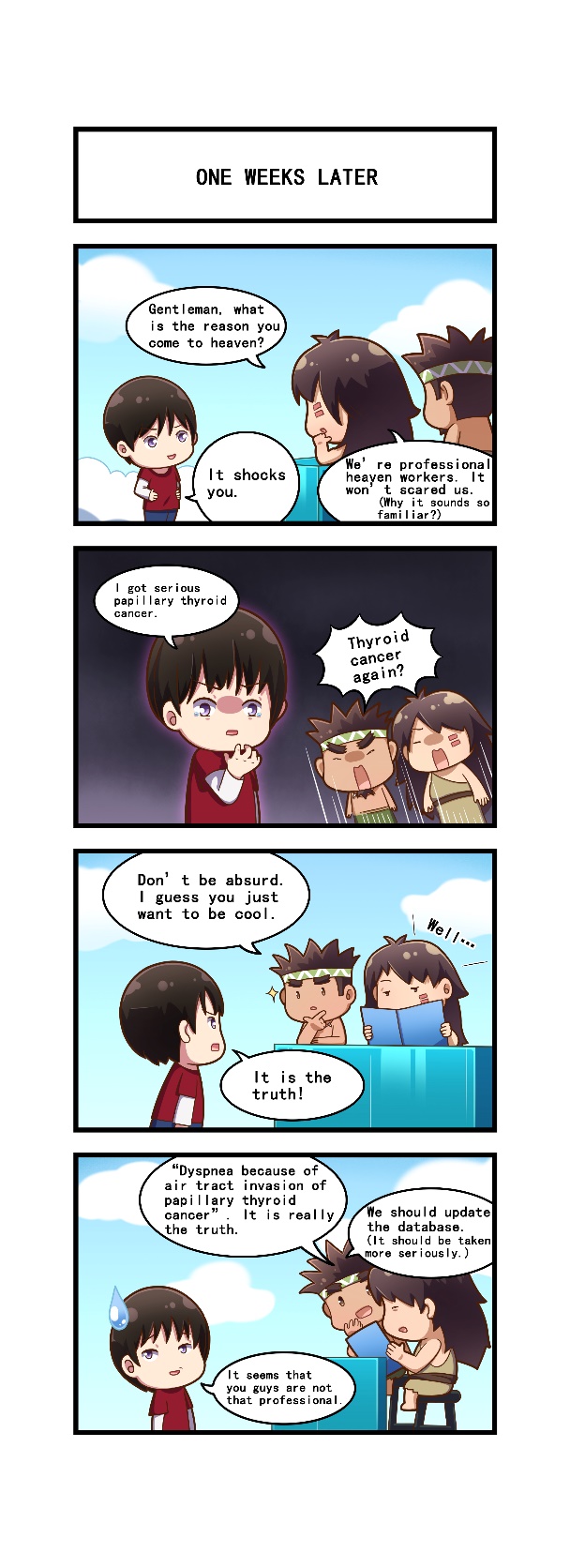


Full comic link (Chinese version): https://mp.weixin.qq.com/s/417VdSHYXqr4ZM1RU419Gw

The comic is mainly talking about two new heaven visitors’ conversation with workers, talking about the cause of death when they arrived in heaven. It is followed by a “Doctor’s Mini-Class,” which features a dialogue between Dr. Yunjian Zhang and Nurse Xiaomeng, introducing knowledge related to papillary thyroid cancer (PTC).

Two different situations show the characteristics of papillary thyroid cancer, which is common type but can be serious.

PTC, might be unfamiliar to the public. Actually, it is the most common type of thyroid cancer, accounting for approximately 90% of all thyroid cancers. It can occur at any age, especially in children and women younger than 40.

Generally, surgery combined with regular drug-taking regimen is enough for many cancer patients. Radioactive iodine therapy is only needed in patients with higher staging, distant metastasis, or extra-glandular invasion involving esophagus or trachea.

Although PTC is very common, it should not be underestimated. Serious PTC can cause hoarseness if it invades the outer boarder and affects the recurrent laryngeal nerve; cause Horner’s syndrome if it affects the sympathetic nerve, cause dysphagia if it involves esophagus, cause dyspnea or even apnea if it affects the airway, and might cause tumor thrombosis in major cervical vessels, etc. Therefore, great attention should be paid. Please visit a thyroid specialist if you found something unusual.

**Supplementary Figure 3. Example of patient health education: a video** (used with permission under a CC BY open access license)


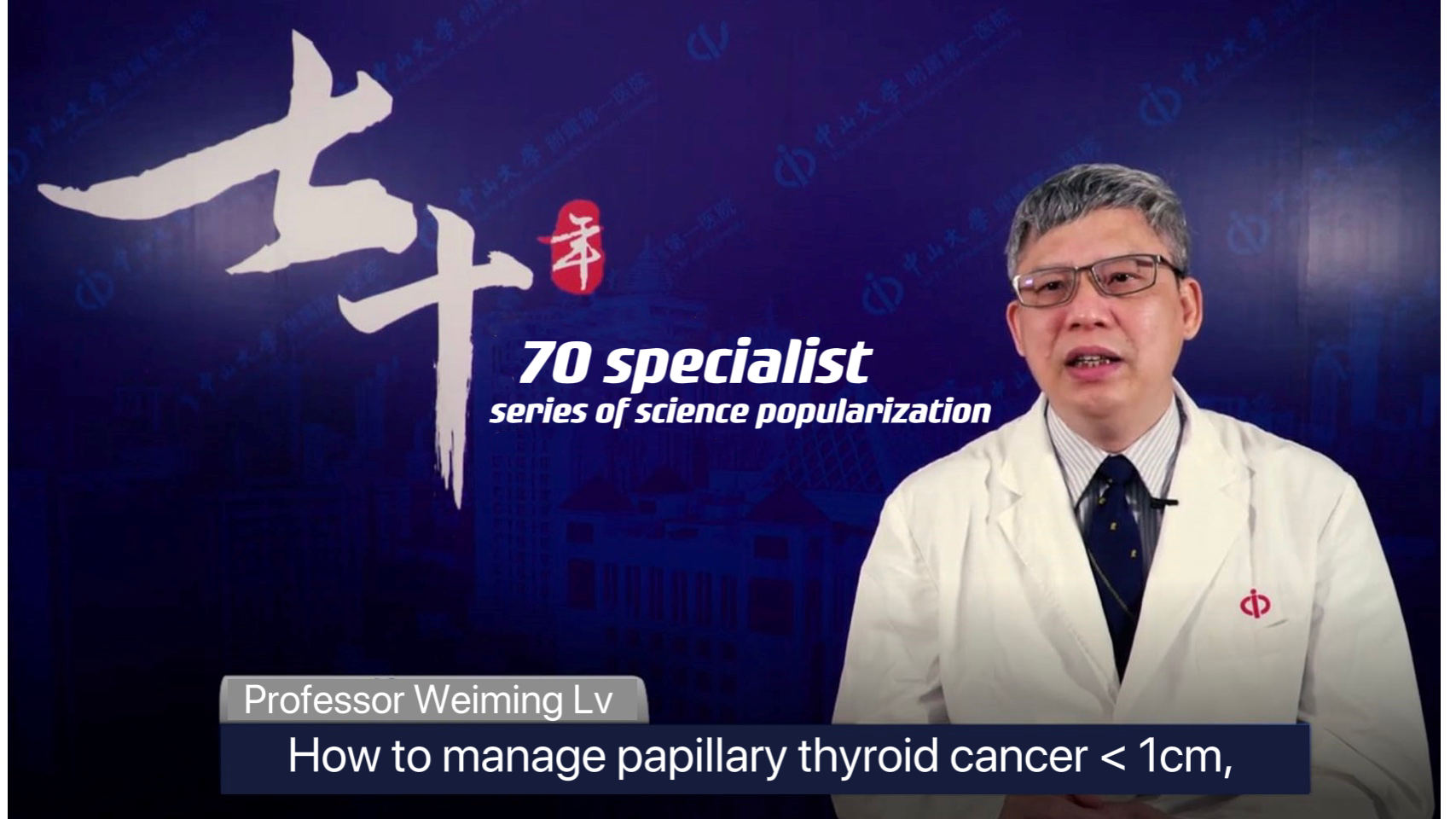


(Screenshot of patient health education in video form in English translation)

Full video link (Chinese version): https://mp.weixin.qq.com/s/V8-0UYJjnZuti86Ff1Qyag

In this video, Weiming Lv (listed author), the director of the Department of Thyroid and Breast Surgery of the First Affiliated Hospital of Sun Yat-sen University, talked about 3 common questions about thyroid.
Firstly, thyroid cancer is not directly related to iodized salt. Iodine stimulates thyroxine production and does not cause nodule formation.
Secondly, most thyroid nodules are benign. Even if your nodule is diagnosed as thyroid cancer, you don't need to be terrified since more than 95% of nodules are papillary thyroid cancer (a highly differentiated subtype) and the 10-year survival rate reached 95-98%.
Thirdly, you should visit a surgeon to acquire accurate therapeutic strategies.

.

**Supplementary Table 1.** Demographics of Questionnaire Respondents (different amount of patient health education)

|  | 0 | 1-10 | 11-20 | >20 | *P-*values |
| --- | --- | --- | --- | --- | --- |
| Age | 38.2 (10.9) | 37.5 (10.4) | 38.4 (9.6) | 37.0 (9.6) | 0.596* |
| Gender (n, %) |  |  |  |  | 0.927 |
| Male | 24 (20.3) | 38 (18.9) | 29 (20.6) | 25 (21.9) |  |
| Female | 94 (79.7) | 163 (81.1) | 112 (79.4) | 89 (78.1) |  |
| Employment status (n, %) |  |  |  |  | 0.356 |
| Employed/Full-time student | 97 (82.2) | 174 (86.6) | 122 (86.5) | 103 (90.4) |  |
| Unemployed/Retired | 21 (17.8) | 27 (13.4) | 19 (13.5) | 11 (9.6) |  |
| Race (n, %) |  |  |  |  | 0.380 |
| Han | 116 (98.3) | 200 (99.5) | 138 (97.9) | 111 (97.4) |  |
| Others | 2 (1.7) | 1 (0.5) | 3 (2.1) | 3 (2.6) |  |
| Relationship status (n, %) |  |  |  |  | 0.670 |
| Married or long-term relationship | 90 (76.3) | 164 (81.6) | 115 (81.6) | 92 (80.7) |  |
| Single, divorced, or widowed | 28 (23.7) | 37 (18.4) | 26 (18.4) | 22 (19.3) |  |
| Highest level of education (n, %) |  |  |  |  | 0.000 |
| Below bachelor’s degree | 71 (60.2) | 82 (40.8) | 59 (41.8) | 27 (23.7) |  |
| Bachelor’s degree or above | 47 (39.8) | 119 (59.2) | 82 (58.2) | 87 (76.3) |  |
| Extent of thyroidectomy (n, %) |  |  |  |  | 0.030 |
| Total thyroidectomy | 48 (40.7) | 82 (40.8) | 62 (44.0) | 65 (57.0) |  |
| Lobectomy | 70 (59.3) | 119 (59.2) | 79 (56.0) | 49 (43.0) |  |
| Extent of neck dissection (n, %) |  |  |  |  | 0.966 |
| No neck dissection | 47 (39.8) | 87 (43.3) | 54 (38.3) | 44 (38.6) |  |
| Ipsilateral lymph nodes dissection | 51 (43.2) | 84 (41.8) | 64 (45.4) | 53 (46.5) |  |
| Bilateral lymph nodes dissection | 20 (16.9) | 30 (14.9) | 23 (16.3) | 17 (14.9) |  |
| Pathological diagnosis (n, %) |  |  |  |  | 0.343 |
| Papillary | 116 (98.3) | 200 (99.5) | 137 (97.2) | 113 (99.1) |  |
| Follicular | 2 (1.7) | 1 (0.5) | 4 (2.8) | 1 (0.9) |  |
| Microcarcinoma (n, %) |  |  |  |  | 0.528 |
| No | 75 (63.6) | 141 (70.1) | 91 (64.5) | 73 (64.0) |  |
| Yes | 43 (36.4) | 60 (29.9) | 50 (35.5) | 41 (36.0) |  |
| TNM staging (n, %) |  |  |  |  | 0.739 |
| I | 117 (99.2) | 199 (99.0) | 138 (97.9) | 113 (99.1) |  |
| II | 1 (0.8) | 2 (1.0) | 3 (2.1) | 1 (0.9) |  |
| RAI (n, %) |  |  |  |  | 0.687 |
| No | 104 (88.1) | 173 (86.1) | 124 (87.9) | 95 (83.3) |  |
| Yes | 14 (11.9) | 28 (13.9) | 17 (12.1) | 19 (16.7) |  |

RAI: radioactive Iodine ablation

*: Not conform to bivariate normal distribution. Non-parametric test is used and data were uniformly described by mean (SD).

**Supplementary Table 2.** Demographics of Questionnaire Respondents (articles)

|  | No | Yes | *P-*values |
| --- | --- | --- | --- |
| Age | 36.0 (8.1) | 38.0 (10.4) | 0.286* |
| Gender (n, %) |  |  | 0.006 |
| Male | 4 (6.8) | 112 (21.7) |  |
| Female | 55 (93.2) | 403 (78.3) |  |
| Employment status (n, %) |  |  | 0.112 |
| Employed/Full-time student | 47 (79.7) | 449 (87.2) |  |
| Unemployed/Retired | 12 (20.3) | 66 (12.8) |  |
| Race (n, %) |  |  | 0.608 |
| Han | 59 (100.0) | 506 (98.3) |  |
| Others | 0 (0.0) | 9 (1.7) |  |
| Relationship status (n, %) |  |  | 0.392 |
| Married or long-term relationship | 45 (76.3) | 416 (80.8) |  |
| Single, divorced, or widowed | 14 (23.7) | 99 (19.2) |  |
| Highest level of education (n, %) |  |  | 0.000 |
| Below bachelor’s degree | 39 (66.1) | 200 (38.8) |  |
| Bachelor’s degree or above | 20 (33.9) | 315 (61.2) |  |
| Extent of thyroidectomy (n, %) |  |  | 0.005 |
| Total thyroidectomy | 16 (27.1) | 241 (46.8) |  |
| Lobectomy | 43 (72.9) | 274 (53.2) |  |
| Extent of neck dissection (n, %) |  |  | 0.279 |
| No neck dissection | 25 (42.4) | 207 (40.2) |  |
| Ipsilateral lymph nodes dissection | 29 (49.2) | 223 (43.3) |  |
| Bilateral lymph nodes dissection | 5 (8.5) | 85 (16.5) |  |
| Pathological diagnosis (n, %) |  |  | 0.582 |
| Papillary | 58 (98.3) | 508 (98.6) |  |
| Follicular | 1 (1.7) | 7 (1.4) |  |
| Microcarcinoma (n, %) |  |  | 0.563 |
| No | 37 (62.7) | 343 (66.6) |  |
| Yes | 22 (37.3) | 172 (33.4) |  |
| TNM staging (n, %) |  |  | 1.000 |
| I | 59 (100.0) | 508 (98.6) |  |
| II | 0 (0.0) | 7 (1.4) |  |
| RAI (n, %) |  |  | 0.157 |
| No | 55 (93.2) | 441 (85.6) |  |
| Yes | 4 (6.8) | 74 (14.4) |  |

RAI: radioactive Iodine ablation

*: Not conform to bivariate normal distribution. Non-parametric test is used and data were uniformly described by mean (SD).

**Supplementary Table 3.** Demographics of Questionnaire Respondents (comics)

|  | No | Yes | *P-*values |
| --- | --- | --- | --- |
| Age | 39.4 (10.8) | 36.4 (9.4) | 0.002* |
| Gender (n, %) |  |  | 0.076 |
| Male | 63(23.5) | 53(17.3) |  |
| Female | 205(76.5) | 253(82.7) |  |
| Employment status (n, %) |  |  | 0.513 |
| Employed/Full-time student | 232 (84.4) | 270 (87.1) |  |
| Unemployed/Retired | 43 (15.6) | 40 (12.9) |  |
| Race (n, %) |  |  | 0.512 |
| Han | 265 (98.9) | 300 (98.0) |  |
| Others | 3 (1.1) | 6 (2.0) |  |
| Relationship status (n, %) |  |  | 0.000 |
| Married or long-term relationship | 232 (86.6) | 229 (74.8) |  |
| Single, divorced, or widowed | 36 (13.4) | 77 (25.2) |  |
| Highest level of education (n, %) |  |  | 0.007 |
| Below bachelor’s degree | 128 (47.8) | 111 (36.3) |  |
| Bachelor’s degree or above | 140 (52.2) | 195 (63.7) |  |
| Extent of thyroidectomy (n, %) |  |  | 1.000 |
| Total thyroidectomy | 120 (44.8) | 137 (44.8) |  |
| Lobectomy | 148 (55.2) | 169 (55.2) |  |
| Extent of neck dissection (n, %) |  |  | 0.717 |
| No neck dissection | 112 (41.8) | 120 (39.2) |  |
| Ipsilateral lymph nodes dissection | 117 (43.7) | 135 (44.1) |  |
| Bilateral lymph nodes dissection | 39 (14.6) | 51 (16.7) |  |
| Pathological diagnosis (n, %) |  |  | 1.000 |
| Papillary | 264 (98.5) | 302 (98.7) |  |
| Follicular | 4 (1.5) | 4 (1.3) |  |
| Microcarcinoma (n, %) |  |  | 0.930 |
| No | 178 (66.4) | 202 (66.0) |  |
| Yes | 90 (33.6) | 104 (34.0) |  |
| TNM staging (n, %) |  |  | 0.260 |
| I | 263 (98.1) | 304 (99.3) |  |
| II | 5 (1.9) | 2 (0.7) |  |
| RAI (n, %) |  |  | 0.807 |
| No | 233 (86.9) | 263 (86.0) |  |
| Yes | 35 (13.1) | 43 (14.1) |  |

RAI: radioactive Iodine ablation

*: Not conform to bivariate normal distribution. Non-parametric test is used and data were uniformly described by mean (SD).

**Supplementary Table 4.** Demographics of Questionnaire Respondents (videos)

|  | No | Yes | *P-*values |
| --- | --- | --- | --- |
| Age | 37.8 (10.3) | 37.8 (10.2) | 0.871* |
| Gender (n, %) |  |  | 0.715 |
| Male | 11 (22.0) | 105 (20.0) |  |
| Female | 39 (78.0) | 419 (80.0) |  |
| Employment status (n, %) |  |  | 0.832 |
| Employed/Full-time student | 43 (86.0) | 453 (86.5) |  |
| Unemployed/Retired | 7 (14.0) | 71 (13.5) |  |
| Race (n, %) |  |  | 1.000 |
| Han | 50 (100.0) | 515 (98.3) |  |
| Others | 0 (0.0) | 9 (1.7) |  |
| Relationship status (n, %) |  |  | 0.580 |
| Married or long-term relationship | 42 (84.0) | 419 (80.0) |  |
| Single, divorced, or widowed | 8 (16.0) | 105 (20.0) |  |
| Highest level of education (n, %) |  |  | 0.177 |
| Below bachelor’s degree | 16 (32.0) | 223 (42.6) |  |
| Bachelor’s degree or above | 34 (68.0) | 301 (57.4) |  |
| Extent of thyroidectomy (n, %) |  |  | 0.882 |
| Total thyroidectomy | 23 (46.0) | 234 (44.7) |  |
| Lobectomy | 27 (54.0) | 290 (55.3) |  |
| Extent of neck dissection (n, %) |  |  | 0.596 |
| No neck dissection | 17 (34.0) | 215 (41.0) |  |
| Ipsilateral lymph nodes dissection | 25 (50.0) | 227 (43.3) |  |
| Bilateral lymph nodes dissection | 8 (16.0) | 82 (15.6) |  |
| Pathological diagnosis (n, %) |  |  | 0.520 |
| Papillary | 49 (98.0) | 517 (98.7) |  |
| Follicular | 1 (2.0) | 7 (1.3) |  |
| Microcarcinoma (n, %) |  |  | 0.159 |
| No | 38 (76.0) | 342 (65.3) |  |
| Yes | 12 (24.0) | 182 (34.7) |  |
| TNM staging (n, %) |  |  | 1.000 |
| I | 50 (100.0) | 517 (98.7) |  |
| II | 0 (0.0) | 7 (1.3) |  |
| RAI (n, %) |  |  | 0.665 |
| No | 42 (84.0) | 454 (86.6) |  |
| Yes | 8 (16.0) | 70 (13.4) |  |

RAI: radioactive Iodine ablation

*: Not conform to bivariate normal distribution. Non-parametric test is used and data were uniformly described by mean (SD).

**Supplementary Table 5.** Multifactor linear regression analysis of amount of PHE and EQRCT QLQ-C30, THYCA-QoL, and HADS scales

| Variables | Coef. | 95% CI | *P-*values |
| --- | --- | --- | --- |
| Global QOL | 1.52 | (0.03, 3.01) | 0.046 |
| Physical† | 0.65 | (-0.62, 1.93) | 0.313 |
| Role† | 1.20 | (-0.17, 2.57) | 0.086 |
| Emotional† | 1.72 | (0.39, 3.05) | 0.011 |
| Cognitive† | 1.76 | (0.02, 3.50) | 0.048 |
| Social† | 0.55 | (-0.69, 1.79) | 0.384 |
| Fatigue* | -0.70 | (-2.37, 0.97) | 0.411 |
| Nausea/vomiting* | -0.14 | (-0.37, 0.09) | 0.223 |
| Pain* | 0.28 | (-1.18, 1.75) | 0.705 |
| Dyspnea | -0.75 | (-2.55, 1.05) | 0.414 |
| Insomnia | -0.58 | (-2.40, 1.23) | 0.529 |
| Appetite loss | -0.87 | (-1.95, 0.21) | 0.115 |
| Constipation | -0.09 | (-0.79, 0.62) | 0.810 |
| Diarrhea | 0.24 | (-0.34, 0.82) | 0.419 |
| Financial | -0.07 | (-1.40, 1.26) | 0.916 |
| Neuromuscular | 0.05 | (-1.21, 1.31) | 0.941 |
| Voice | -0.19 | (-1.79, 1.41) | 0.814 |
| Concentration | -1.12 | (-2.77, 0.52) | 0.181 |
| Sympathetic | -0.63 | (-2.32, 1.06) | 0.462 |
| Throat/mouth | -0.04 | (-1.44, 1.35) | 0.950 |
| Psychological | -0.89 | (-2.34, 0.55) | 0.226 |
| Sensory | -0.95 | (-2.56, 0.66) | 0.249 |
| Scar | 2.43 | (0.19, 4.67) | 0.033 |
| Chilly | -1.53 | (-2.93, -0.13) | 0.032 |
| Tingling | 0.95 | (-0.57, 2.48) | 0.221 |
| Weight gain | -0.33 | (-2.38, 1.72) | 0.753 |
| Headache | -1.05 | (-2.98, 0.89) | 0.288 |
| Sex | 1.35 | (-0.51, 3.21) | 0.156 |
| Anxiety | -0.25 | (-0.53, 0.02) | 0.073 |
| Depression | -0.34 | (-0.62, -0.06) | 0.017 |

Note: The baseline factors were adjusted including sex, age, occupation, ethnicity, marital status, educational level, extent of thyroidectomy and lymph node dissection, paraffin pathological diagnosis, paraffin diagnosed as microcarcinoma, TNM staging and radioactive Iodine ablation.

†Higher scores indicate better functioning (functional domains); *Higher scores indicate more symptoms (symptom domains).

**Supplementary Table 6.** Multifactor linear regression analysis of articles and EQRCT QLQ-C30, THYCA-QoL, and HADS scales

| Variables | Coef. | 95% CI | *P-*values |
| --- | --- | --- | --- |
| Global QOL | -3.39 | (-8.44, 1.67) | 0.189 |
| Physical† | 2.71 | (-1.60, 7.01) | 0.217 |
| Role† | -0.03 | (-4.68, 4.61) | 0.989 |
| Emotional† | -1.93 | (-6.46, 2.61) | 0.404 |
| Cognitive† | -0.60 | (-6.52, 5.31) | 0.841 |
| Social† | -3.05 | (-7.25, 1.16) | 0.155 |
| Fatigue* | 0.55 | (-5.11, 6.21) | 0.849 |
| Nausea/vomiting* | -0.42 | (-1.19, 0.35) | 0.287 |
| Pain* | 3.74 | (-1.22, 8.69) | 0.139 |
| Dyspnea | -3.56 | (-9.66, 2.53) | 0.251 |
| Insomnia | 5.43 | (-0.71,11.56) | 0.083 |
| Appetite loss | -4.11 | (-7.75, -0.46) | 0.027 |
| Constipation | 1.32 | (-1.06, 3.70) | 0.276 |
| Diarrhea | 0.26 | (-1.70, 2.23) | 0.792 |
| Financial | -0.60 | (-5.09, 3.89) | 0.794 |
| Neuromuscular | 1.51 | (-2.76, 5.77) | 0.488 |
| Voice | -0.45 | (-5.86, 4.97) | 0.872 |
| Concentration | 1.46 | (-4.12, 7.03) | 0.607 |
| Sympathetic | 2.56 | (-3.16, 8.27) | 0.380 |
| Throat/mouth | 0.45 | (-4.26, 5.16) | 0.851 |
| Psychological | 2.42 | (-2.49, 7.33) | 0.333 |
| Sensory | 4.93 | (-0.52,10.38) | 0.076 |
| Scar | 2.34 | (-5.27,9.94) | 0.546 |
| Chilly | 2.67 | (-2.09, 7.42) | 0.271 |
| Tingling | 2.10 | (-3.07, 7.27) | 0.425 |
| Weight gain | 0.69 | (-6.24, 7.62) | 0.846 |
| Headache | 0.44 | (-6.11, 6.99) | 0.894 |
| Sex | -1.03 | (-7.35, 5.28) | 0.748 |
| Anxiety | 0.11 | (-0.83, 1.06) | 0.815 |
| Depression | -0.08 | (-1.03, 0.87) | 0.865 |

Note: The baseline factors were adjusted including sex, age, occupation, ethnicity, marital status, educational level, extent of thyroidectomy and lymph node dissection, paraffin pathological diagnosis, paraffin diagnosed as microcarcinoma, TNM staging and radioactive Iodine ablation.

†Higher scores indicate better functioning (functional domains); *Higher scores indicate more symptoms (symptom domains).

**Supplementary Table 7.** Multifactor linear regression analysis of comics and EQRCT QLQ-C30, THYCA-QoL, and HADS scales

| Variables | Coef. | 95% CI | *P-*values |
| --- | --- | --- | --- |
| Global QOL | 2.32 | (-0.72, 5.35) | 0.135 |
| Physical† | 0.81 | (-1.78, 3.40) | 0.540 |
| Role† | 1.68 | (-1.11, 4.47) | 0.238 |
| Emotional† | 0.10 | (-2.63, 2.82) | 0.945 |
| Cognitive† | 1.46 | (-2.09, 5.02) | 0.419 |
| Social† | 2.96 | (0.44, 5.48) | 0.021 |
| Fatigue* | -1.62 | (-5.02, 1.78) | 0.349 |
| Nausea/vomiting* | -0.35 | (-0.82, 0.11) | 0.138 |
| Pain* | 0.89 | (-2.10, 3.87) | 0.559 |
| Dyspnea | -1.66 | (-5.32, 2.01) | 0.375 |
| Insomnia | -4.98 | (-8.66, -1.31) | 0.008 |
| Appetite loss | 1.13 | (-1.07, 3.33) | 0.316 |
| Constipation | -1.09 | (-2.52, 0.34) | 0.134 |
| Diarrhea | 0.04 | (-1.14, 1.22) | 0.943 |
| Financial | -3.18 | (-5.87, -0.49) | 0.020 |
| Neuromuscular | -2.18 | (-4.74, 0.38) | 0.095 |
| Voice | -2.47 | (-5.72, 0.78) | 0.136 |
| Concentration | -2.31 | (-5.65, 1.04) | 0.177 |
| Sympathetic | -1.78 | (-5.22, 1.65) | 0.309 |
| Throat/mouth | -2.64 | (-5.46, 0.19) | 0.067 |
| Psychological | -1.75 | (-4.70, 1.20) | 0.244 |
| Sensory | -1.01 | (-4.30, 2.28) | 0.546 |
| Scar | -10.08 | (-14.58, -5.58) | 0.000 |
| Chilly | -2.91 | (-5.77, -0.06) | 0.045 |
| Tingling | -0.30 | (-3.41, 2.81) | 0.850 |
| Weight gain | -0.84 | (-5.00, 3.33) | 0.694 |
| Headache | -3.40 | (-7.34, 0.53) | 0.090 |
| Sex | 0.29 | (-3.51, 4.09) | 0.881 |
| Anxiety | 0.09 | (-0.48, 0.66) | 0.763 |
| Depression | 0.17 | (-0.41, 0.74) | 0.567 |

Note: The baseline factors were adjusted including sex, age, occupation, ethnicity, marital status, educational level, extent of thyroidectomy and lymph node dissection, paraffin pathological diagnosis, paraffin diagnosed as microcarcinoma, TNM staging and radioactive Iodine ablation.

†Higher scores indicate better functioning (functional domains); *Higher scores indicate more symptoms (symptom domains).

**Supplementary Table 8.** Multifactor linear regression analysis of videos and EQRCT QLQ-C30, THYCA-QoL, and HADS scales

| Variables | Coef. | 95% CI | *P-*values |
| --- | --- | --- | --- |
| Global QOL | -1.03 | (-6.33, 4.27) | 0.703 |
| Physical† | -2.53 | (-7.04, 1.97) | 0.270 |
| Role† | -0.17 | (-5.03, 4.69) | 0.947 |
| Emotional† | -0.73 | (-5.47, 4.01) | 0.763 |
| Cognitive† | 1.83 | (-4.36, 8.02) | 0.563 |
| Social† | -0.38 | (-4.78, 4.03) | 0.866 |
| Fatigue* | -0.19 | (-6.11, 5.73) | 0.951 |
| Nausea/vomiting* | -0.46 | (-1.27, 0.35) | 0.263 |
| Pain* | -0.40 | (-5.60, 4.79) | 0.879 |
| Dyspnea | -1.17 | (-7.55, 5.21) | 0.718 |
| Insomnia | -0.28 | (-6.72, 6.15) | 0.931 |
| Appetite loss | 1.20 | (-2.63, 5.03) | 0.539 |
| Constipation | -0.07 | (-2.56, 2.42) | 0.955 |
| Diarrhea | -0.79 | (-2.84, 1.27) | 0.451 |
| Financial | -0.03 | (-4.74, 4.67) | 0.988 |
| Neuromuscular | -3.11 | (-7.57, 1.35) | 0.171 |
| Voice | -1.48 | (-7.15, 4.18) | 0.607 |
| Concentration | -1.95 | (-7.78, 3.89) | 0.512 |
| Sympathetic | 0.84 | (-5.14, 6.83) | 0.782 |
| Throat/mouth | -3.60 | (-8.52, 1.31) | 0.151 |
| Psychological | -1.55 | (-6.69, 3.59) | 0.554 |
| Sensory | 1.55 | (-4.17, 7.27) | 0.594 |
| Scar | -4.54 | (-12.49, 3.41) | 0.263 |
| Chilly | 2.81 | (-2.17, 7.78) | 0.268 |
| Tingling | -2.49 | (-7.91, 2.92) | 0.366 |
| Weight gain | -0.69 | (-7.94, 6.56) | 0.852 |
| Headache | -2.19 | (-9.04, 4.66) | 0.530 |
| Sex | -1.27 | (-7.88, 5.34) | 0.707 |
| Anxiety | -0.22 | (-1.21, 0.76) | 0.656 |
| Depression | -0.04 | (-1.04, 0.96) | 0.938 |

Note: The baseline factors were adjusted including sex, age, occupation, ethnicity, marital status, educational level, extent of thyroidectomy and lymph node dissection, paraffin pathological diagnosis, paraffin diagnosed as microcarcinoma, TNM staging and radioactive Iodine ablation.

†Higher scores indicate better functioning (functional domains); *Higher scores indicate more symptoms (symptom domains).
